# Pain Neuroscience Education in Children and Adolescents with Chronic Pain: A Systematic Review

**DOI:** 10.3390/children12101317

**Published:** 2025-10-01

**Authors:** Mónica Pico, Carmen Matey-Rodríguez, Ana Domínguez-García, Noemí Yubero, Alejandro Santos-Lozano

**Affiliations:** 1 i+HeALTH Strategic Research Group, Department of Health Sciences, Miguel de Cervantes European University (UEMC), 47012 Valladolid, Spain; cmatey@uemc.es (C.M.-R.); adominguez@uemc.es (A.D.-G.); nyubero@uemc.es (N.Y.); asantos@uemc.es (A.S.-L.); 2 Research Institute of the Hospital 12 de Octubre (‘imas12’), 28041 Madrid, Spain

**Keywords:** pain neuroscience education, chronic pain, pediatrics, children, adolescents, self-efficacy, function, emotional functioning, biopsychosocial model

## Abstract

**Highlights:**

**What are the main findings?**

- Pain neuroscience education improves pain understanding, functionality, and self-efficacy in children and adolescents with chronic pain.
- Pain neuroscience education also influences emotional variables such as anxiety, catastrophizing, and kinesiophobia, although effects are often modest and short-lived.

**What is the implication of the main finding?**

- Combining pain neuroscience education with physical exercise and implementing school- or digital-based delivery formats may enhance effectiveness and broaden the reach of interventions.
- Including parents and caregivers in pain neuroscience education programs could improve clinical outcomes and support a more comprehensive and sustainable approach to pediatric chronic pain.

**Abstract:**

**Background/Objectives:** Pain neuroscience education (PNE) has demonstrated efficacy in adults with chronic pain, but the pediatric evidence is still developing, despite its increasingly frequent use. Evidence for the effectiveness of PNE in pediatrics remains fragmented across settings and outcomes, which justifies a systematic evaluation focused on children and adolescents. **Methods:** Following PRISMA, two reviewers independently screened records (PubMed, Web of Science, PEDro; through 21 July 2025), extracted data, and assessed risk of bias (RoB 2 for randomized controlled trials; NIH/CASP for non-randomized studies). Given the heterogeneity, we conducted a structured narrative synthesis (SWiM) and rated the certainty of evidence with GRADE. PROSPERO: CRD420251062922. **Results:** Eleven studies met the inclusion criteria. PNE consistently improved pain-related knowledge, with effects maintained at follow-up (moderate certainty); effects on pain intensity, function, and emotional outcomes were small and inconsistent (low certainty), with more favorable patterns when PNE was combined with exercise and/or booster sessions. Digital and gamified formats proved feasible and engaging; parental outcomes showed small improvements where measured. **Conclusions:** PNE is a promising, low-cost, and scalable component of pediatric chronic pain care, strengthening self-efficacy and adaptive coping. Integration into biopsychosocial, multidisciplinary programs—particularly alongside exercise and family involvement—may optimize outcomes. Larger, standardized trials with long-term follow-up and systematic adverse-event reporting are needed to solidify guidance for clinical practice.

## 1. Introduction

Pain is an unpleasant experience associated with actual or potential tissue damage, involving sensory, emotional, cognitive, and social components [1]. Chronic pain (CP) is defined as pain that persists for more than three months after the initial injury or cause has resolved and therefore lacks the acute warning function of physiological nociception, constituting a condition in its own right [2]. In accordance with ICD-11 in pediatrics, we distinguish: (a) chronic primary pain—pain in one or more anatomical regions, lasting ≥3 months, associated with significant emotional distress and/or functional disability, not better accounted for by another condition; and (b) chronic secondary pain—pain attributable to an underlying disease or condition (e.g., postsurgical/post-traumatic, neuropathic, headache disorders, visceral disorders). Recurrent acute pain refers to time-limited episodes that recur but do not meet the ≥3-month duration and/or the disorder-specific frequency thresholds required for a chronic diagnosis (e.g., episodic migraine or recurrent abdominal pain below chronicity criteria) [1].

Recent population-based estimates indicate that approximately 20.8% of children and adolescents report chronic or recurrent pain (CP)—equivalent to 1 in 5—according to the recent review by Chambers et al. [3], noting that the prevalence in their study refers to the population proportion reporting CP symptoms rather than clinical diagnoses. Among the different types of PCP the prevalence of headache is 25.7%, back pain 19.1%, abdominal pain 17.3%, musculoskeletal pain 25.7%, multisite/generalized pain 21.0%, and other types of pain 6.9% [3]. It is also noteworthy that girls have a higher prevalence of CP (18.3%) compared to boys (12.7%) [3]. Evidence suggests that lower socioeconomic development, and in some cases sociocultural factors, contribute to the increasing incidence of CP [4].

However, CP does not affect all children and adolescents in the same way. Traditional definitions of CP do not always consider the degree of interference it causes in daily life. For this reason, the concept of “high-impact chronic pain” (HICP) has been proposed, aiming to identify patients who experience significant dysfunction or functional limitations associated with CP [5]. It is estimated that approximately 7% of the pediatric population experiences HICP, which is characterized by a level of intensity and persistence sufficient to substantially limit daily activities [5].

Although the etiology of PCP remains unclear, it has been associated with physical [6,7] psychosocial [8], and contextual factors [9,10,11]. Its pathophysiology is complex and involves mechanisms such as central sensitization (CS), in which inhibitory pain pathways fail, cerebral synapses are altered, and pain perception is amplified [12,13,14,15]. However, due to pediatric neuroplasticity, findings in adults cannot be directly extrapolated to children [16,17].

CP affects children beyond physical discomfort, with consequences that are both multiple and severe. These include physical and functional limitations [18], sleep disturbances [19,20], psychological disorders [21,22], school bullying [23], academic absenteeism, and even brain alterations [24]. PCP pain is a strong predictor of CP in adulthood, making childhood pain a significant risk factor for long-term chronification [25,26]. This may perpetuate a cycle of ongoing disability and reduced quality of life [27]. Furthermore, PCP often disrupts family functioning, as parents and siblings commonly face increased stress, financial strain, and interruptions in daily routines [27]. The economic burden associated with PCP is also considerable [27,28].

Traditionally, treatment approaches have focused on pharmacological and physical interventions, yet these have demonstrated significant limitations [29]. In this context, pain neuroscience education (PNE) has emerged as a promising alternative, centered on teaching children and their families how the brain modulates the pain experience [30]. PNE promotes a more objective understanding of pain, reduces fear, and encourages shifts in beliefs and attitudes, ultimately leading to improved functionality and quality of life [31].

Although recent years have seen substantial efforts to improve the diagnosis and management of pediatric pain [32,33], PCP remains a prevalent health concern. PNE has accumulated supportive evidence in adults, and current international guidance [32] emphasizes biopsychosocial, multimodal care with education for children and families. However, structured PNE is not yet systematically integrated into pediatric services, and its effectiveness in this population requires clarification. This review aims to synthesize the pediatric evidence on the effectiveness of PNE in the management of pediatric chronic pain to inform clinical implementation and research priorities.

## 2. Materials and Methods

This overview of a systematic literature review was conducted following the guidelines established in the Preferred Reporting Items for Systematic Reviews and Meta-Analyses (PRISMA) statement [34], the complete checklist is available in Appendix A, Table A1.

### 2.1. Systematic Review Protocol Registration

In accordance with international recommendations and to ensure the highest standards of transparency and integrity, the protocol for this overview of systematic reviews was registered in the International Prospective Register of Systematic Reviews (PROSPERO) database (https://www.crd.york.ac.uk/prospero/, accessed on 17 August 2025), administered by the National Institute for Health Research, under the protocol number CRD420251062922 prior to initiating the literature search. There were no amendments.

### 2.2. Eligibility Criteria

Studies aligned with the primary objective of describing the effectiveness of ENP interventions for PCP. Specifically, they included studies that met the following criteria: (1) randomized trials, quasi-experimental studies, and single-group pre-post studies that evaluated (2) PNE interventions in (3) clinical or school pediatric population with CP (primary and secondary). Studies were excluded if pain was procedural, oncological, associated with a primary neurological condition or recurrent acute pain. No restrictions were placed on the publication date to ensure a comprehensive collection of relevant literature. The complete list of excluded full-text reports, with citation and main reason, is provided in Appendix B, Table A2.

### 2.3. Design and Search Strategy

To ensure a comprehensive and methodical approach, an advanced search strategy was designed and implemented across the following electronic databases: PubMed, Web of Science (WOS), and PEDro. The search concluded on 21 July 2025.

**Figure A1:**
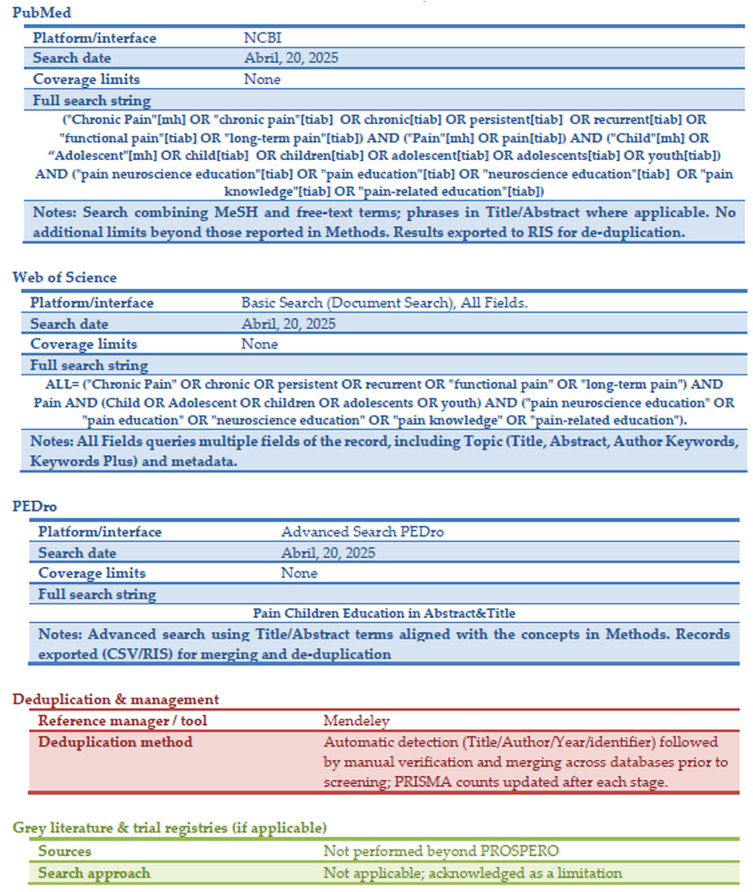
Full Electronic Search Strategies.

The search strategy was based on keywords, free-text terms, and Medical Subject Headings (MeSH), systematically combined using the Boolean operators AND/OR. The search strings were tailored to each database’s syntax and are reported in full in Appendix C, Figure A1. This approach enabled the identification and retrieval of relevant evidence, ensuring comprehensive and rigorous coverage of the literature.

### 2.4. Data Extraction

A total of 230 articles were identified. Duplicates were removed through automatic checking and manual review. No automation tools were used for screening. Two reviewers (MP and AD) worked independently at all stages. Study data were extracted using a standardized Excel form (version 2020), which was tested and revised as necessary. Full-text selection was performed independently by the same two reviewers (MP and AD). Any conflicts regarding study inclusion were resolved through discussion. A third reviewer (CM) was consulted to reach consensus on the final selection of included studies. We contacted original authors when eligibility was unclear, particularly regarding the content of the PNE intervention, or to request full-text articles. Forward and backward citation tracking of included studies was also performed to identify additional relevant publications.

For each included study, we extracted the following characteristics: study design and setting; participants’ age and sex; diagnosis and pain location within the context of CP. We also recorded details of the intervention and comparator, including the delivery modality of PNE, intervention duration, and follow-up length. Regarding outcomes, we collected all prespecified variables at all reported time points (post-intervention and follow-ups), prioritizing validated pediatric instruments (PedIMMPACT). Prespecified outcomes of interest were: (1) critical (pain knowledge, pain intensity, disability/function, Patient Global Impression of Change (PGIC), and adverse events); (2) important (catastrophizing, kinesiophobia, sleep, self-efficacy, coping, CS, depression, medication use, satisfaction, and parent-related variables.) When applicable, we considered parent-involved interventions and extracted their outcomes.

### 2.5. Study Quality and Assessment of Risk of Bias

Two reviewers (MP and NY) independently assessed the risk of bias for each outcome at each time point, using design-specific tools. Disagreements were resolved by consensus or, if necessary, by a third reviewer (AD).

For randomized controlled trials (RCTs) and pilot studies, the Cochrane Risk of Bias 2 (RoB2) tool was applied, which evaluates five domains: randomization process, deviations from intended interventions, missing outcome data, outcome measurement, and selection of the reported result. Each domain is rated as “low risk,” “some concerns,” or “high risk,” producing an overall risk of bias judgment [35].

The qualitative study was assessed using the Critical Appraisal Skills Programme (CASP) checklist, consisting of 10 questions aimed at appraising the validity, relevance, and methodological rigor of qualitative research [36].

The feasibility study was evaluated with the NIH Study Quality Assessment Tools (official checklist; last up-dated July 2021), which include design-specific checklists to assess methodological clarity, validity of measurements, and transparency in reporting [37].

We assessed certainty of evidence using the outcome-level GRADE approach for narrative syntheses (no meta-analysis). We prespecified the following as critical outcomes: pain knowledge, pain intensity, disability/function, PGIC, and adverse events; and as important outcomes: catastrophizing, kinesiophobia, sleep, self-efficacy, pain coping, CS, depression, medication use, satisfaction with treatment/care, and parental behaviors/family accommodation (e.g., HHI-Pain). Because this is a pediatric context, we stratified by informant (child/adolescent self-report vs. parent/caregiver self-report). When a child outcome was measured via parent proxy, we considered potential indirectness and downgraded certainty when appropriate. Downgrading decisions considered risk of bias, inconsistency, indirectness, imprecision, and publication bias. Two reviewers rated independently and resolved disagreements by consensus. Summary of Findings (SoF) tables and Evidence Profiles by informant are presented in Appendix D (Table A3, Table A4 and Table A5).

### 2.6. Synthesis and Effect Measures

For each domain, studies were grouped by comparator (NE alone, NE + exercise or other conditions), setting (school, clinic, digital) and population. If a study provided multiple measures for the same domain, the validated instructions were preferred, and in case of redundancy, the predefined primary measure was preferred. A specific assessment of bias due to missing results (e.g., effects of small studies) was not performed.

Given heterogeneity in interventions, comparators, outcome measures, and follow-up timing, we conducted a structured narrative synthesis following the SWiM [38] reporting guideline, summarizing direction and magnitude of effects by domain, precision, and synthesis limitations; certainty was rated with GRADE.

## 3. Results

### 3.1. Search Results

**Figure 1:**
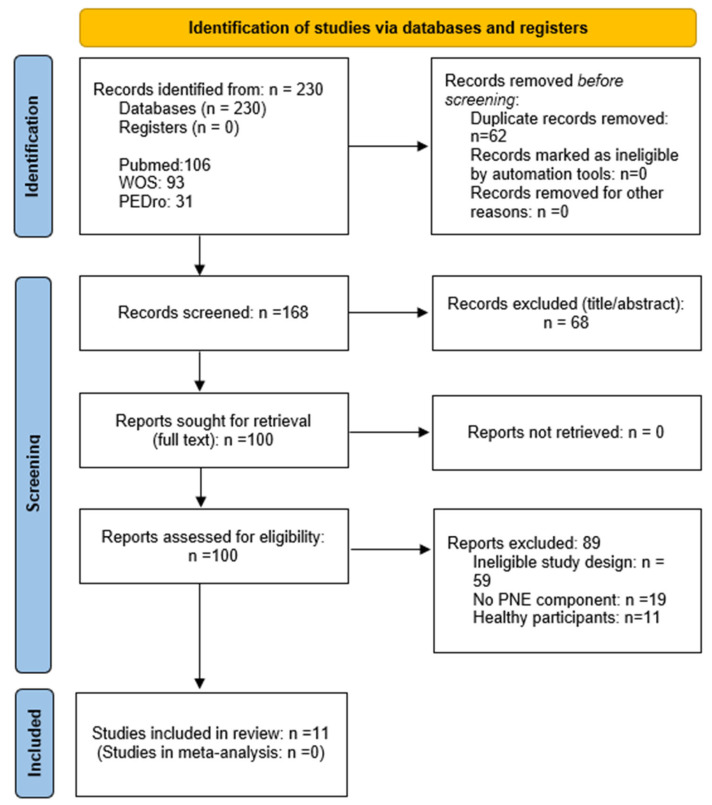
PRISMA diagram summarizing search results.

The study selection process is depicted in the PRISMA flow diagram (Figure 1). A total of 230 records were identified (PubMed = 106; Web of Science = 93; PEDro = 31). After removing 62 duplicates, 168 titles/abstracts were screened. One hundred full-text reports were evaluated. Eleven studies were included and 89 were excluded; the main reasons were ineligible design (*n* = 58), no PNE component (*n* = 20), and healthy participants (*n* = 11). The complete list of full-text exclusions is presented in Appendix B, Table A2.

### 3.2. Study Quality and Risk-of-Bias Assessment

Most of the randomized controlled trials and pilot studies included in this review [39,40,41,42,43,44,45,46,47] were rated using the Cochrane RoB 2 tool (official Excel implementation, 22 August 2019 version) [35]; most were judged “low risk of bias” across several domains, with remaining concerns noted as appropriate.

Note: The RoB 2 tool [35] can be applied to randomized pilot trials; however, results should be interpreted with caution, considering that these studies primarily focus on evaluating feasibility and procedures rather than estimating treatment effects, which may influence the ratings obtained.

The qualitative study [48], assessed using the CASP checklist [36], met most methodological criteria, demonstrating clear objectives, appropriate methodology, rigorous data collection, ethical approval, and valuable results. Minor limitations were identified in the researcher–participant relationship, which could introduce some bias, but overall the study was rated as “excellent”.

The feasibility study [49], evaluated using the NIH Quality Assessment Tool (official checklist; last up-dated July 2021) [37], received an overall rating of “acceptable quality”. Its strengths included a clearly defined objective, consistent application of the intervention, appropriate statistical methods, and an acceptable follow-up rate. Limitations were related to incomplete representativeness of the study population, lack of repeated outcome assessments, and partial control of potential confounding variables.

All information related to study quality can be found in Appendix D (Table A3, Table A4 and Table A5).

Regarding the certainty of evidence using the GRADE approach, in children/adolescents (self-report), pain knowledge showed improvements with moderate certainty (7 studies; Appendix E, Table A6 and Table A8). Disability/function (4 studies) and pain intensity (6 studies) showed small or mixed effects, with low certainty (Appendix E, Table A6 and Table A8). PGIC indicated perceived post-treatment improvement (1 study; low; Appendix E, Table A6 and Table A8). Satisfaction with treatment/care was high to moderate but heterogeneous across studies (low; Appendix E, Table A6 and Table A8. Other outcomes (catastrophizing, kinesiophobia, sleep, self-efficacy, pain coping, CS, depression, medication use) showed small or inconclusive effects with very low certainty (1–2 studies each; Appendix E, Table A6 and Table A8). Adverse events: Not reported (Appendix E, Table A6).

In parents/caregivers (self-report), parental catastrophizing (PCS-P) decreased with low certainty (1 study; Appendix E, Table A7 and Table A9); caregiver anxiety/stress showed small or non-sustained effects (very low; Appendix E, Table A7 and Table A9); and parental behaviors/family accommodation (e.g., HHI-Pain) decreased slightly with heterogeneity (low; Appendix E, Table A7 and Table A9). For parent-reported proxy outcomes for the child (disability/function and pain intensity), findings were mixed/inconclusive with low certainty (Appendix E, Table A7 and Table A9; downgraded for indirectness; see Appendix E, Table A10).

### 3.3. Description of Included Studies

The characteristics of the 11 selected articles are presented in Appendix F, Table A11. Substantial heterogeneity across intervention formats, outcome tools, and follow-up windows limited comparability and precluded pooling.

Most of the studies were clinical trials [39,42,43,44,45,47,48], three were pilot [40,41,46] studies, and one was a feasibility study [49]. Two pre-post studies without controls were identified [46,49].

The majority of the studies were conducted in educational settings where the participants were enrolled [40,42,43,45,47,48,49]. One study was carried out in a university hospital [41], another in a neurology clinic [46], and two were conducted online [39,44].

#### 3.3.1. Sample Characteristics

The total sample across the studies consisted of 1076 participants, aged between 6 and 18 years. In all articles reporting sex data [39,40,41,44,46,48,49], the total number of males was 227 and females 467. These figures exclude the study by Neto et al. [48], as it used the sample from Andías et al. [40].

The types of diagnosed CP included headaches [46], abdominal pain [41,44], and cervical pain [40,45,48]. Several articles reported multiple CP, which in addition to the above, included musculoskeletal pain [39,42,43,47,49].

Four studies included parents or caregivers in the research [39,41,44,46].

#### 3.3.2. Type of Intervention

All articles employed PNE as the intervention tool; however, the format of PNE used varied across the studies. Below, the specific types of PNE utilized in each study are described:

PNE delivered through informational sessions via lectures was the most common format, although the studies employing this approach [40,42,45,46,47,48] implemented it in different ways. In the study by Andías et al. [40] and Neto et al. [48], who used the same sample, four educational sessions were conducted over four consecutive weeks in groups of 4–7 participants. Andías et al. [45] expanded the intervention to five sessions, some delivered in-person and others via WhatsApp due to the SARS-CoV-2 pandemic. Participants in both studies received Supplementary Materials in the form of informational brochures.

Louw et al. [42] provided a single 30-min session followed by reinforcement videos at 2 and 4 months to review the content. Menés et al. [47] also included reinforcement; their PNE program consisted of two sessions—the first lasting 90 min and the second 60 min, delivered one month later. This program additionally incorporated gamification and guided methods (problem-based learning), which are described under other types of interventions later.

In all the studies described above, except Louw et al. [42], the sessions were delivered by researchers experienced in the subject. In Louw et al. [42], the sessions were conducted by teachers trained by physicians as well as by the physicians themselves.

The study by Beach et al. [46] also included an educational session, but in this case, it was conducted following an interview with participants about their experience and understanding of pain, and lasted 10 min. During this session, a 3D brain model was used as a visual aid, focusing on pain processing and sensitization, as well as the biopsychosocial factors influencing pain control. The session concluded with a discussion on neuroplasticity and learning, emphasizing the importance of training the brain through education and physical activity (PA) to experience less pain.

In the study by Menés et al. [47], a second session was developed based on a problem-solving activity involving group dynamics where participants had to classify different situations as either dangerous or not to the integrity of the body. The main goal of this session was to define tissue damage and reduce the threat value of various situations related to non-tissue damage explanations.

PNE delivered through a film was addressed in the studies by Wager et al. [49] and Kisling et al. [43]. Both studies used an 11-min educational film titled “Understanding pain—and what’s to be done about it in 10 min,” available on YouTube. This educational film was developed by researchers at the German Pediatric Pain Center in 2014 and is based on existing evidence regarding the development of chronic pain and strategies for its management. Participants viewed the video during school hours.

Two studies [41,47] used gamification as a strategy for delivering PNE. Pas et al. [41] utilized the game PNE4Kids, developed based on the book “Explaining Pain” by Butler and Moseley [50]. This material employs a military analogy to explain to children and their families how the pain system works, its changes in response to persistent pain, and how to apply this knowledge to their personal experience. In the study by Pas et al. [41], the experimental group (EG) underwent an initial hypnosis session followed by a group-based PNE session, in which the PNE4Kids program was combined with a hypnosis session. In the study by Menés et al. [47], a role-playing game was used in which two groups of participants (12–14 people each) represented brain pain processing through a business metaphor involving two different individuals. Each group was divided into subteams with specific roles tasked with evaluating whether their actions would cause pain in the person they represented across three scenarios (two involving tissue damage and one without). The decisions were recorded on a Control Panel and discussed after each scenario.

PNE delivered via the Internet was conducted in the studies by Palermo et al. [39] and Walker et al. [44]. In these studies, the PNE group served as the control group, while the intervention group received cognitive behavioral therapy (CBT) also delivered online. Both treatment groups had access to two different versions of a web-based program, the Web-based Management of Adolescent Pain program (Web-MAP2), which offered either CBT or PNE. The control version of the Web-MAP website included two functional components: (1) modules containing information compiled from publicly available educational websites on pediatric chronic pain management, and (2) a diary and assessments. The control website did not provide access to behavioral or cognitive skills training. Adolescents and their parents were instructed to log into the web program weekly, following the same schedule as the CBT group, to read information about PPC, with reminders sent every two weeks. In Walker et al.’s study [44], the program was the same but modified to specifically address functional abdominal pain (FAPD).

**Figure 2:**
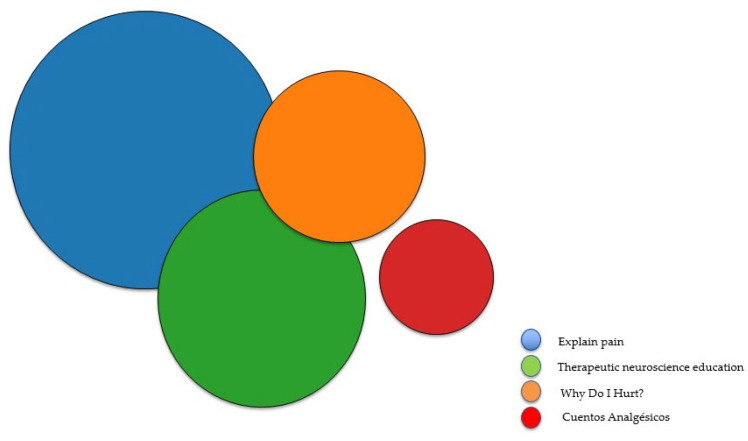
Sources of PNE used in the studies; bubble size represents the number of studies citing each source. Explaining Pain [50]; Therapeutic neuroscience education [51]; Why Do I Hurt? [53]; Cuentos analgésicos [54].

In all studies, the content of the PNE programs aligned with the key concepts of PNE interventions for children and adolescents, following the international guidelines outlined by Butler and Moseley [50] and Louw and Puentedura [51]. Core topics included the neurophysiology of pain, the transition from acute to chronic pain, and the capacity of the nervous system to modulate the pain experience [52]. CS was explicitly mentioned in the studies by Andías et al. [45] and Louw et al. [42]. Andías et al. [45] also addressed the importance of exercise, beliefs, emotions, and sleep, while Louw et al. [42] additionally described various endogenous therapeutic strategies for pain relief. Figure 2 and Table S1 presents the sources used in each of the studies.

Regarding the control groups (CG), Andías et al. [40], Neto et al. [48], and Beach et al. [46] did not implement any intervention in their CG. Andías et al. [45] included general and functional exercises that involved full-body loading as well as more specific exercises targeting the neck and shoulder regions. Menés et al. [47] reported that the CG maintained their usual school curriculum program. In the study by Pas et al. [41], the CG group received two sessions of hypnotherapy, which is the standard care FAPD in the country where the study was conducted (Belgium).

Regarding the follow-up periods conducted in each study, the following conclusions can be drawn: the studies by Andías et al. [40], Neto et al. [48], and Wager et al. [49] did not include any follow-up after the intervention. Pas et al. [41] conducted follow-up at 3 weeks, while Kisling et al. [43] performed follow-up at 4–5 weeks. Menés et al. [47] conducted follow-ups at 7 and 13 weeks. Palermo et al. [39], Louw et al. [42], Andías et al. [45], and Beach et al. [46] conducted follow-ups at 6 months. The longest follow-up was conducted by Walker et al. [44], with assessments at 6 and 12 months.

#### 3.3.3. Assessment Tools and Outcomes Obtained

Various outcome measures were employed across the studies to evaluate multiple dimensions related to PCP. These included assessments of pain intensity, modulation, frequency, and localization, as well as muscular function. Emotional functioning was measured through anxiety and kinesiophobia scales. Additionally, participants’ pain knowledge, functional ability (including disability, pain-related helplessness, and self-efficacy), sleep quality, and factors influencing pain modulation were evaluated. Quality of life and treatment satisfaction were also reported. Behavioral changes related to pain coping strategies and medication use were monitored throughout the interventions. In studies involving parents or caregivers, emotional functioning (anxiety, protective behaviors, and pain-related helplessness) and treatment satisfaction were also assessed. All outcome assessments were quantitative, with the exception of Neto et al. [48], which conducted a qualitative evaluation focusing on the relevance of acquired knowledge and participants’ perceptions of the intervention.

##### Pain Intensity and the Severity of Pain-Related Symptoms

Were assessed in 6 of the 11 studies reviewed [39,40,41,43,44,45]. The instruments used to assess pain intensity included the NRS-11 [39], VAS [40], FPS-R [41], NPRS [45] and NRS [43]. It should be noted that the terms NRS, NPRS, and NRS-11 all refer to the same 11-point numerical scale (0–10) for assessing pain intensity. Symptom severity was assessed only in Walker et al. [44], using the Gastrointestinal (GI) Symptoms Subscale of the CSSI-24 and the Abdominal Pain Index (API). No significant improvements in pain intensity were observed in Palermo et al. [39], Andías et al. [40], or Kisling et al. [43]. In contrast, Pas et al. [41] and Andías et al. [45] reported significant reductions that were maintained at follow-up. Walker et al. [44] found no between-group differences (intervention vs. control) when all participants were analyzed together; however, both groups showed significant improvements in symptoms. Notably, this RCT conducted a subgroup analysis based on baseline symptom severity, revealing that participants with more severe symptoms experienced significantly greater reductions in GI symptoms during the treatment period and at follow-up compared to the PNE control condition.

##### Pain Modulation

Since pain modulation influences the perception and experience of pain, one study evaluated symptoms associated with CS. In this context, the Central Sensitization Inventory (CSI) is used to quantify such symptoms. Andías et al. [45] were the only authors to include this variable in their study. The CSI consists of 25 items and is scored from 0 to 100, with higher scores indicating a greater presence of CS symptoms. Their study reported mean scores of 35.04 in the EG and 37.76 in the CG, corresponding to a mild level of symptoms according to the standard interpretation of the scale.

##### Emotional Function

Four emotional domains were assessed across the included studies: anxiety, depression, catastrophizing, and kinesiophobia.

Anxiety was evaluated in 2/11 studies. Andías et al. [40] used the State-Trait Anxiety Inventory for Children (STAIC), comprising state and trait anxiety subscales. No statistically significant between-group differences were reported, although the experimental group showed a reduction in scores. Palermo et al. [39] applied the Bath Adolescent Pain Questionnaire (BAPQ), combining three highly correlated subscales (depression, general anxiety, and pain-related anxiety) into a single valid and reliable composite score. Internet-delivered cognitive behavioral therapy (CBT) produced modest improvements in emotional functioning post-intervention, with small but statistically significant reductions in depressive symptoms and pain-related anxiety compared to the PNE control group. These effects were not maintained at the 6-month follow-up.

Catastrophizing was assessed in two studies by Andías et al. [40,45] using the Pain Catastrophizing Scale (PCS). Both reported decreased scores, reaching statistical significance only in the 2022 study [45].

Kinesiophobia was measured in two studies. Louw et al. [42] used the Fear-Avoidance Beliefs Questionnaire—Physical Activity (FABQ-PA), while Andías et al. [45] employed the Tampa Scale for Kinesiophobia (TSK). Both reported improvements in both intervention and control groups, with statistical significance observed only in Andías et al. [45].

##### Pain-Related Knowledge

Seven studies assessed knowledge or conceptual understanding of pain using six instruments: the Neurophysiology of Pain Questionnaire (NPQ) [40,45] and its revised version rNPQ [42], the Impact of Pain Questionnaire (COPI) [46], the Pediatric Pain Knowledge Questionnaire—Children’s Version (PKQ-CH) [43,49], and the Child and Adolescent Pain Conceptualization Questionnaire (COPAQ) [47].

The NPQ (13 items) assesses knowledge of pain neurophysiology and is particularly useful in the context of PNE programs. The COPI (14 items) measures the perceived impact of pain on functional, emotional, and social domains, adopting a biopsychosocial framework for clinical intervention. The COPAQ (15 items) and PKQ-CH (20 items), specifically developed for pediatric populations, assess pain conceptualization and factual knowledge, respectively.

All studies evaluating pain-related knowledge or conceptualization in pediatric populations [40,42,43,45,46,47,49] reported positive findings, with statistically significant increases post-intervention, maintained at follow-up.

##### Functionality

Several studies assessed functionality in children and adolescents with CP using different instruments: the Pediatric Pain Disability Index (PPDI) [43], the Child Activity Limitations Interview (CALI) [39], the PROMIS Pediatric Pain Interference—Short Form 8a [44], and a custom questionnaire measuring pain-related behavior change [42].

The FDI and IED-P scales, both based on 15 items, and the PPDI (7 items) measure functional disability perceived by the child or caregivers; the CALI assesses limitations in activities selected by the adolescent; and the PROMIS evaluates pain interference across social, cognitive, emotional, and physical domains.

The results show reductions in functional disability across several studies, although statistical significance was reached only in some cases. In the study by Palermo et al. [39], online CBT produced greater reductions in activity limitations at six months compared to online PNE, although no differences were observed immediately after treatment. Walker et al. [44] reported significant improvements in both groups in pain interference, with no differences between them. In contrast, Kisling et al. [43] and Louw et al. [42] found no significant changes in the functional outcomes assessed. Overall, improvements were modest, with effect sizes varying according to the measurement instrument and the timing of the assessment.

##### Sleep

Two studies assessed sleep quality in children and adolescents with CP [39,45]. Palermo et al. [39] used the 10-item short version of the Adolescent Sleep–Wake Scale (ASWS-10), validated in youth with CP. Internet-delivered CBT (EG) produced a slightly greater improvement in sleep quality at 6 months compared to the PNE group (CG), although this difference was not statistically significant immediately post-treatment. In contrast, Andías et al. [45] employed the Basic Scale of Insomnia Complaints and Sleep Quality (BaSIQS), finding significant sleep quality improvements in both groups through to the final follow-up.

##### Pain Coping

Pain coping was assessed in three studies [39,43,45] using different instruments: the Helping for Health Inventory adapted for CP (HHI-Pain) [39], the Pediatric Pain Coping Inventory—revised (PPCI-r) [43], and the Child Self-Efficacy Scale (CSES) [45].

The HHI-Pain measures perceived social support related to pain management by identifying behaviors that may facilitate or hinder pain coping. Palermo et al. [39] reported a significant improvement in this variable at 6 months post-intervention, suggesting increased positive social support and facilitative strategies for pain management.

The PPCI-r evaluates specific pain coping strategies employed by children, including cognitive, emotional, and behavioral aspects. Kisling et al. [43] found no significant changes on this scale, indicating that the intervention did not produce relevant modifications in the coping strategies assessed.

Finally the CSES assesses the self-efficacy perceived by children and adolescents to cope with pain and maintain daily functioning despite it. In the study by Andías et al. [45], significant improvements in self-efficacy were observed in both groups up to follow-up, indicating increased confidence in managing pain adaptively.

Together, these findings suggest that certain dimensions of pain coping, such as social support and self-efficacy, may improve following interventions, whereas others, like specific coping strategies, may be more resistant to change.

##### Medication Use

Medication use was assessed in only one study [42], which reported a significant reduction in medication consumption during the subsequent school year in the group that received PNE with follow-up reinforcement.

##### Satisfaction

Satisfaction with the intervention was assessed using the Treatment Evaluation Inventory—Short Form (TEI-SF) in the studies by Palermo et al. [39] and Walker et al. [44], with significantly higher scores reported in the EG. Satisfaction with changes in health status was measured using the Patient Global Impression of Change (PGIC) in both studies by Andías et al. included in this review [40,45], showing a significant perception of improvement in the EG in both cases.

In the qualitative study by Neto et al. [48], participants reported the acquired knowledge as a facilitator of changes in feelings, attitudes, and behaviors toward pain, including reduced anxiety, fear, and catastrophizing, as well as increased self-efficacy to manage it. The intervention was well received by all participants, perceived as useful, and considered to have appropriate materials and strategies.

##### Parental Assessment in the Included Studies

Four studies considered the participation and evaluation of parents regarding pain management in children and adolescents [39,41,44,46] and only one evaluated outcomes beyond 12 months [44]. The variables assessed included emotional and behavioral aspects, as well as satisfaction with the intervention, using various validated instruments.

Palermo et al. [39] measured parental emotional functioning (BAPQ-PIQ), protective behavior (Protect subscale of ARCS), perceived failed help (HHI-Pain), and satisfaction with the intervention (TEI-SF). They found a significant reduction in protective behavior in the CG and greater satisfaction with the intervention in the EG, with no significant changes in perceived failed help. Additionally, they reported that internet-based CBT (EG) led to improvements in parental emotional impact, reducing anxiety, depression, maladaptive behaviors, and self-blame.

Pas et al. [41] assessed parental catastrophizing (PCS-P), fear of pain (FOPQ-P), and functionality (FDI-P), observing significant improvements in both the EG and CG after the intervention and at follow-up.

Walker et al. [44] only assessed parental satisfaction with the intervention (TEI-SF), which was significantly higher in the EG, while Beach et al. [46] did not apply specific instruments to parents, although they were present during the intervention.

## 4. Discussion

The primary aim of this review was to explore the potential of PNE as a therapeutic intervention in children and adolescents with CP, providing evidence on its effectiveness in reducing pain and improving functionality. Research on the use and value of PNE in pediatric chronic pain remains limited; however, the available studies demonstrate notable efficacy. PNE-based interventions have shown improvements in pain understanding, facilitated reconceptualization of pain, reduced fear of movement, and enhanced functionality in children. These findings underscore the need to further investigate this approach, given its therapeutic and preventive potential during a critical stage of development.

The earliest study included was published in 2016 [39], while the most recent dates from 2025 [47], reflecting the increasing scientific interest in this field over the past decade. Several investigations corresponded to pilot trials [40,41,46] and feasibility studies [49], which primarily assessed feasibility, acceptability, and preliminary effects of PNE in pediatric populations. Although such designs provide valuable insights to guide future research, their exploratory nature limits the ability to draw firm conclusions about effectiveness and does not represent high-level evidence according to methodological standards. Nevertheless, their inclusion is relevant, given the scarcity of RCTs in this field and the pressing need to consolidate a robust body of evidence regarding educational interventions for PCP.

### 4.1. Study Quality and Risk of Bias

When interpreting these findings, study quality and risk of bias warrant consideration. Overall, methodological quality was heterogeneous, which limits the interpretation of effects. Several randomized trials and pilot studies showed low risk of bias in key RoB-2 domains (e.g., randomization process, missing outcome data, outcome measurement, and reporting), yet ratings of “some concerns” or “high risk” were frequent in critical areas such as deviations from intended interventions—a common challenge for educational interventions in real-world settings, outcome measurement, the use of non-validated scales or unblinded assessment, and selection of the reported result. These patterns can overestimate short-term effects and support GRADE downgrades for risk of bias, inconsistency, and imprecision. Small sample sizes and limited follow-up increased imprecision, while variability in instruments and comparators contributed to inconsistency across studies.

Qualitative and feasibility studies supported content validity and acceptability (overall CASP appraisals “excellent”; NIH ratings “acceptable quality”), but showed expected limitations (non-representative samples, absence of repeated measurements, lack of blinding) that restrict external validity and causal inference. For parent-reported proxy outcomes of the child (e.g., disability/function and pain), indirectness was present and certainty was downgraded accordingly in GRADE. Non-reporting of adverse events limited safety appraisal despite this being a pre-specified critical outcome. Taken together, these considerations help explain why, apart from pain knowledge (moderate certainty), overall certainty was low or very low for most outcomes.

High or unclear risk in outcome measurement and selective reporting, together with limited blinding for self-reported outcomes, likely contributed to the inconsistent patterns observed for pain intensity and emotional outcomes. In addition, although a formal assessment of publication bias was not feasible given the small number of studies per outcome, small-study effects and selective reporting cannot be ruled out. Accordingly, future research should: (1) preregister protocols and outcomes; (2) use validated pediatric measures (e.g., the PedIMMPACT core outcome set); (3) implement blinded outcome assessment whenever feasible; (4) ensure adequate sample sizes and consider cluster designs where appropriate; (5) standardize PNE content and dose; (6) monitor and systematically report adverse events; and (7) report outcomes stratified by informant, prioritizing child self-report for subjective outcomes. These steps will reduce bias, heterogeneity, and imprecision, thereby increasing certainty and clinical translation.

### 4.2. Sample and Setting

Regarding the sample, the studies included children and adolescents aged 6–18 years, with a predominance of adolescents. According to Louw et al. [42], students aged 11–13 years are better equipped to understand complex concepts, which may enhance the impact of PNE; this age range was represented across all studies in the present review. In most trials [39,40,41,44,48], a higher proportion of female participants was reported, consistent with evidence showing that CP is at least twice as prevalent among females, who also present a higher risk of developing it and increased sensitivity to certain painful stimuli [55,56]. This female predominance in PCP samples has important implications for both research and clinical practice. On one hand, it underscores the need to further investigate the biological, psychological, and sociocultural mechanisms related to sex that may contribute to differences in pain perception and persistence. On the other hand, it suggests that PNE programs for adolescents should incorporate gender-sensitive approaches. Tailoring interventions to these needs could enhance their effectiveness and support a more equitable management of PCP.

A notable feature of this review is the frequent use of small samples [40,41,46,48,49]. Although smaller groups allow individualized interventions and facilitate follow-up, in a multidimensional phenomenon such as pediatric chronic pain, sufficient sample variability is necessary to obtain representative and robust results; studies with small sample sizes tend to overestimate effects and to yield inconsistent findings when replicated [57]. Consequently, although valuable in initial stages, future trials should recruit larger and more diverse samples in terms of age, sex, and socio-educational context to enhance statistical power and the clinical and educational applicability of their findings.

Another relevant aspect of the sample is the inclusion of parents and/or caregivers in neuroscience-based educational interventions targeting PCP. Only four studies [39,41,44,46] considered their participation, and in one of them [46], caregivers were included solely as companions. This low level of involvement is striking given the fundamental role of the family environment in managing PCP [58,59]. Previous theoretical models [42,60,61] highlight the bidirectional relationship between child experiences and family responses and recommend integrating these variables into intervention design. PNE offers an opportunity to simultaneously address cognitive and emotional processes in both parents and children, promoting more empathetic and less overprotective responses. Findings from this review suggest that systematically including caregivers could provide valuable insights into emotional, behavioral, and perceptual changes and should be prioritized in future research.

Regarding the setting, it is noteworthy that six of the eleven studies were conducted in schools [40,42,43,47,48,49]. Schools play a key role in health literacy, as they are environments where children and adolescents spend a significant portion of their time [49]. This makes schools an ideal context for implementing preventive programs and supporting students who have not yet developed a CP condition, helping to prevent dysfunctional functioning and pain chronification [49]. However, recent studies indicate that knowledge of PCP in school settings is generally limited, leading to misunderstanding and inadequate support for affected students [62]. This lack of teacher training may contribute to the invisibility of PCP and the absence of effective intervention strategies in schools. Therefore, structured educational interventions that include both teachers and students are necessary, not only to improve the quality of life of affected students but also to foster a more inclusive and supportive school environment.

### 4.3. Formats to Deliver PNE

The interventions included in this review employed various formats to deliver PNE to pediatric populations, all sharing a common core: the transmission of content related to the neuroscience of pain. The evidence suggests that repeated or booster sessions enhance its impact [42,47], consistent with the theory of repeated exposure learning [63,64]. Among complementary strategies, some studies incorporated gamification [41,47], problem-solving based on personal experiences [47], and internet-based programs [39,44], which yielded improved outcomes, albeit not across all variables. Digital tools and mobile apps may be particularly advantageous for adolescents who are familiar with virtual environments, although their success depends on improvements in design, usability, and clinical integration [65,66]. Digital environments additionally facilitate population-level reach, enabling large-scale interventions. Another promising technology is virtual reality (VR), which was not utilized in the reviewed studies but has demonstrated benefits for pain perception and management [67] and the neurofunctional rehabilitation, inducing neuroplastic changes in the brain, particularly in regions associated with sensory perception and motor control [68] which could increase the pain thresholds, reduce anxiety [69], and enhance motor function. Its implementation could serve as a complementary tool to PNE interventions. Overall, these modalities—emphasizing active participation, personal experience, and meaningful interaction with content—move away from passive teaching models and show potential for fostering more enduring changes in pain understanding and coping. However, knowledge alone does not guarantee behavior change [70]; therefore, future studies should evaluate not only knowledge acquisition but also its translation into adaptive behaviors and the reduction in pain-related disability.

In this review, only three studies—conducted by the same research group—combined PNE with PA as an integral part of treatment [40,45,48], reporting positive outcomes. Therapeutic exercise is strongly supported by the scientific literature for the management of PCP [71]. Beyond its effect on pain, PA contributes to mental health by reducing anxiety, depression, and fear of pain—factors frequently associated with PCP [72]—and may foster healthier lifestyle habits when introduced early in life. However, the evidence for the effectiveness of physical activity (PA) in reducing pain intensity and improving disability and quality of life, compared with usual care, is of very low certainty [73]. This suggests that, while exercise is beneficial, its effectiveness could be enhanced when combined with educational interventions such as PNE, breaking the fear–avoidance cycle and promoting physical recovery and psychological adaptation.

### 4.4. Outcome Measures and Assessment Tools

Regarding the variables assessed, several issues can be addressed in this discussion. With respect to the outcome measures employed, only two studies [39,45] adhered to the recommendations of the *Pediatric Initiative on Methods, Measurement, and Pain Assessment in Clinical Trials* (PedIMMPACT), which defines the core outcome domains for pediatric pain clinical trials [74]. Although pain intensity is a commonly used outcome in PCP research, the findings of this review indicate that it does not always accurately reflect the impact of PNE. Only two [41,45] of the six studies that evaluated it [39,40,41,43,44,45] reported significant and sustained reductions. This pattern supports the notion that PNE exerts its strongest influence on cognitive, emotional, and behavioral aspects rather than on the sensory-discriminative dimension of pain. Moreover, pain intensity is a volatile measure influenced by transient factors such as mood, PA, or social context, which may hinder the detection of intervention-related changes. The study by Walker et al. [44] further reinforces this perspective, showing that only participants with more severe baseline symptoms achieved significant improvements, suggesting that initial severity modulates treatment response. Therefore, and consistent with a biopsychosocial framework, future studies should prioritize multidimensional measures such as functional disability, quality of life, and coping strategies, as these may more accurately capture the broader and more sustainable effects of PNE in pediatric populations.

Central sensitization (CS), assessed in this review exclusively by Andías et al. [45] using the Central Sensitization Inventory (CSI)—represents a relevant marker of dysfunction in pain modulation. The reported scores correspond to a mild level of symptoms, which may reflect a lower CS burden than that observed in adult chronic pain populations [75]; nevertheless, these patients exhibit dysfunction in descending inhibitory pathways, contributing to pain persistence and vulnerability to chronification [76]. From this perspective, PNE, by fostering pain reconceptualization and reducing fear, may help reactivate inhibitory processes, particularly when combined with therapeutic exercise, psychological approaches, and attentional regulation techniques. Therefore, incorporating a measure of pain-modulation dysfunction to identify subgroups with more pronounced alterations is of interest and could guide personalized interventions.

In the included studies, emotional variables—anxiety, catastrophizing, and kinesiophobia—were repeatedly assessed, but effects were heterogeneous and often modest or not sustained over time [39,40,42,45]. Anxiety and depression are common comorbidities in this population [77] and may exacerbate functional impairment; concern about pain and feelings of helplessness account for much of this burden [78], which supports incorporating educational content on emotion regulation, coping, and the neurophysiology of pain to mitigate their impact. Catastrophizing, defined as an excessively negative orientation toward pain, is associated with greater disability and poorer functioning [79] but it can be reduced through PNE and active pain-management strategies. Kinesiophobia is a key barrier to functional recovery and is associated with poorer physical performance and quality of life [79]. In the studies reviewed, both groups improved, although statistical significance was observed only in Andías et al. [45]. Taken together, these findings support a biopsychosocial, individualized approach to pediatric chronic pain, combining PNE, graded PA, and psychological support, and underscore the need for more robust studies with long-term follow-up to determine the sustainability of effects and optimize intervention strategies.

PNE in the pediatric population appears to be effective in improving conceptual understanding of pain, as shown by the seven included studies that assessed this variable [40,42,43,45,46,47,49]. The results suggest significant increases in pain understanding following educational interventions, and these effects were maintained at follow-up, supporting the efficacy of PNE in promoting sustainable cognitive changes. Enhanced pain knowledge may be a key factor in driving behavioral change [50], indeed, an adequate conceptualization enables the child or adolescent to interpret pain more functionally and adjust their coping strategies, fostering self-efficacy and active participation in pain management. Despite positive findings, methodological challenges remain. Some studies used non-validated pediatric questionnaires [40,42], limiting comparability and reliability. The field should converge on validated, age-appropriate instruments (e.g., PKQ-CH, COPAQ) to more accurately evaluate educational programs and enable cross-study comparisons.

Reduced functionality in pediatric patients with CP represents a significant impact on their quality of life, affecting physical, social, and emotional aspects of child development. The included studies show that, although some interventions achieve reductions in functional disability, the effects are generally modest and vary depending on the measurement instrument and timing of assessment [39,42,43,44]. These findings underscore the need for an approach that combines medical, therapeutic, and behavioral interventions, individually tailored [80], with the aim of preventing functional deterioration, promoting healthy development, and improving the child’s autonomy and participation in daily activities.

Sleep disturbances are a key factor in the experience of PCP, they intensify pain perception and negatively affect quality of life and daily functioning; moreover, they tend to be more persistent and severe in children and adolescents with CP [81]. Although this was the least explored domain in the included studies, the available data show improvements following interventions [39,45]. Overall, sleep disturbances constitute a critical component of pediatric chronic pain; incorporating evidence-based strategies—sleep-hygiene education, cognitive-behavioral interventions, and management of nocturnal pain—is essential to optimize clinical outcomes and promote healthy development in this population.

### 4.5. Follow-Up

The studies included in the review presented variable follow-up durations and intervals. Some conducted immediate post-intervention assessments [40,48,49] and short-term follow-ups between 3 and 5 weeks [41,43], while others extended follow-up to six [39,42,45,46] and twelve months [44,47]. This follow-up allowed the evaluation of the durability of the observed effects, showing sustained improvements in aspects such as perceived pain, functionality, and catastrophizing. This limitation is not exclusive to PNE but is common in many CP interventions [80]. Lack of follow-up reduces the external validity of studies and limits the translation of findings into clinical practice; moreover, systematic follow-up is essential not only to prevent the development of chronic pain [82]. but also to monitor central nervous system changes that contribute to increased pain sensitivity (CS) [83]. In this way, ensuring adequate follow-up is critical to avoid pain chronification and to guarantee the long-term sustainability of intervention outcomes.

### 4.6. Implications for Clinical Practice

Management of CP requires a multidisciplinary approach that addresses physical, emotional, and social dimensions. Findings from this review show that more comprehensive interventions achieved better and more durable outcomes in anxiety, depression, and sleep quality, as well as reductions in maladaptive parental responses [39]. Intensive multidisciplinary programs and specialized pediatric pain units offer the best prospects for patients who do not respond to outpatient therapies, improving functioning and development [84]. Therefore, adequately trained multidisciplinary teams and dedicated pediatric pain units are needed to address the current challenges of pediatric chronic pain.

It is important to note the finding by Walker et al. [44], who identified that the subgrouping of GI pain symptoms based on pain-related psychological characteristics acted as a moderator of response to psychological treatment. Very few studies have explored moderators in pediatric pain treatments. A recent exception evaluated various demographic and psychological factors in a mixed group of CP patients, observing that the intervention was more effective in younger adolescents and in those whose parents exhibited lower levels of emotional distress [85]. Similarly, Ceniza-Bordallo et al. [86] proposed a stratified multimodal physiotherapy intervention based on risk using the Pediatric Pain Screening Tool (PPST), showing preliminary evidence of its effectiveness. These findings suggest that the strategic use of subgroup profiles can optimize resource allocation and enhance intervention efficacy, allowing the identification of patients most likely to benefit from targeted treatments for children and adolescents with CP.

Methodological heterogeneity among studies makes it difficult to draw definitive conclusions regarding the optimal duration and frequency of intervention sessions. Differences in the implementation of PNE—including duration, content, and delivery modality—as well as in the characteristics of the CP conditions studied complicate direct comparison of results. This variability represents one of the main challenges in advancing knowledge, prevention, and treatment of PCP. Overcoming these obstacles requires promoting research with rigorous and comparable methodological designs, as well as developing standardized consensuses and protocols tailored to the needs of the pediatric population. Only in this way will it be possible to optimize prevention, diagnosis, and intervention, thereby reducing the impact of CP on the quality of life and functionality of affected children and adolescents.

### 4.7. Limitations

This systematic review has several limitations. Regarding the review process itself, we searched three major databases and did not conduct a systematic search of gray literature or trial registries beyond protocol registration (PROSPERO). No automation tools were used. These decisions may have reduced comprehensiveness and are acknowledged as limitations of the process. With respect to publication bias and small-study effects, although the small number of studies per outcome precluded formal assessments (e.g., funnel plots), small-study effects and selective outcome reporting cannot be ruled out. This risk is heightened by pilot/feasibility designs and incomplete statistical reporting. To mitigate it, future trials should preregister protocols and outcomes, adhere to reporting standards (including null results), and ensure public access to analysis plans and deidentified data. As for the clinical relevance of small changes in pain, overall improvements were small and frequently below the available MCIDs for pediatric instruments. Because group means can mask clinically meaningful improvement in subgroups, we recommend pre-specifying MCIDs, reporting responder analyses (e.g., ≥30% reduction), and linking symptom change to functional gains and patient-prioritized goals (school attendance, sleep, physical activity) to determine whether statistically detectable effects translate into clinically.

### 4.8. Future Research Directions

Based on the findings of this review, several priority areas for future research can be identified. First, adequately powered randomized controlled trials with larger and more representative samples are needed to more accurately evaluate the effectiveness of PNE in pediatric populations. Standardization of intervention protocols and outcome measures would facilitate comparison across studies and strengthen the scientific evidence base. Second, future research should explore the role of caregivers in educational interventions and examine the impact of PNE on family-related emotional and behavioral variables. Incorporating developmentally tailored adaptations is essential: in younger children, concrete language, visual/gamified supports, short sessions, and co-learning with parents may optimize engagement, whereas in adolescents, autonomy-supportive messaging, relevance to daily life (school, sports, sleep), and digital delivery formats (e.g., apps, interactive chatbots, or virtual reality) may enhance learning and adherence. Third, studies should investigate the combined effects of PNE with therapeutic exercise and other multimodal strategies, as well as the use of risk stratification tools to guide personalization of interventions. Finally, incorporating long-term follow-up is fundamental to assess the durability of effects and their impact on preventing pain chronification.

## 5. Conclusions

Pain neuroscience education (PNE) appears promising as a low-cost, scalable component of pediatric chronic pain care, with consistent gains in pain knowledge and small, variable effects on pain intensity, function, and emotional outcomes—findings shaped by small samples, heterogeneous methods, and limited blinding. Pending adequately powered, standardized trials with long-term follow-up, caregiver components, and a priori consideration of moderators, PNE should be implemented as part of multidisciplinary biopsychosocial programs rather than as a stand-alone therapy. Future studies should explicitly evaluate efficacy under controlled conditions and effectiveness in real-world settings, incorporate developmental tailoring, and report adverse events and MCID-based clinical relevance.

## Data Availability

All data relevant to the study are included in the article or uploaded as Supplementary Information.

## Supplementary Materials

The following supporting information can be downloaded at: https://www.mdpi.com/article/10.3390/children12101317/s1, Table S1: Sources of PNE used in the studies.

## Abbreviations

The following abbreviations are used in this manuscript:

CBT	Cognitive Behavioral Therapy
CP	Chronic Pain
CS	Central Sensitization
CSI	Central Sensitization Inventory
EC	Control Group
EG	Experimental Group
FAPD	Functional Abdominal Pain
HICP	High-Impact Chronic Pain
MeSH	Medical Subject Headings
PA	Physical Activity
PCP	Pediatric Chronic Pain
PedIMMPACT	Pediatric Initiative on Methods, Measurement, and Pain Assessment in Clinical Trials
PGIC	Patient Global Impression of Change
PNE	Pain Neuroscience Education
RCTs	Randomized Controlled Trials
VR	Virtual Reality
Web-MAP2	Web-based Management of Adolescent Pain program
WOS	Web of Science

## Appendix A. PRISMA 2020 Checklist

This checklist documents where each PRISMA 2020 item is addressed in the manuscript and its appendices.

**Table A1:** PRISMA 2020 Checklist.

Section/Topic	Item	Checklist Item (Short Description)	Where Reported	Notes
Title	1	Identify the report as a systematic review.	Title page (“…A Systematic Review”)	
Abstract	2	Provide a structured summary per PRISMA 2020 for abstracts.	Abstract (Background/Objectives; Methods; Results; Conclusions; PROSPERO)	
Introduction	3	Rationale—describe the rationale for the review.	Introduction, para 1–3	
Introduction	4	Objectives—provide an explicit statement of the objective(s).	Introduction, last paragraph	
Methods	5	Eligibility criteria—specify inclusion and exclusion criteria.	Section 2.2 Eligibility Criteria	
Methods	6	Information sources—describe all sources (databases, registers, other), dates of last search.	Section 2.3 Design and Search Strategy (PubMed, WOS, PEDro; last search 21 July 2025); 2.4 Data Extraction	
Methods	7	Search strategy—present full search strategies for all databases, including filters/limits.	Appendix C (Full Electronic Search Strategies)	
Methods	8	Selection process—specify how studies were selected, how many reviewers, independence, automation tools.	Section 2.4 Data Extraction (two reviewers; third reviewer for consensus; no automation tools)	
Methods	9	Data collection process—who extracted data and how; any contacting of authors.	Section 2.4 Data Extraction (two reviewers; contacted authors as needed)	
Methods	10a	Data items—list and define all outcomes for which data were sought, specifying if all time points were sought.	Section 2.4 Data Extraction (prespecified outcomes and time points; PedIMMPACT priority)	
Methods	10b	Data items—list and define all other variables for which data were sought (e.g., participant/setting, funding).	Section 2.4 Data Extraction (study characteristics, intervention/comparator, follow-up)	
Methods	11	Study risk of bias assessment—specify methods used to assess risk of bias in included studies.	Section 2.5 Study quality and assessment of risk of bias (RoB-2/CASP/NIH tools)	
Methods	12	Effect measures—specify for each outcome the effect measure(s) used.	Section 2.6 Synthesis and effect measures	Narrative synthesis; effect measures not applicable; reported as in studies.
Methods	13a–f	Synthesis methods—describe methods for analyses and synthesis; handling of data; subgroup/sensitivity analyses; meta-bias.	Section 2.6 Synthesis and effect measures	No meta-analysis planned due to heterogeneity.
Methods	14	Reporting bias assessment—describe methods to assess risk of bias due to missing results (publication bias).	Appendix E (Publication bias columns marked ‘Unclear’ per GRADE)	No formal small-study/publication bias test performed.
Methods	15	Certainty assessment—describe methods used to assess certainty/certainty (e.g., GRADE).	Section 2.5 (GRADE) and Appendix D/E	
Results	16a	Study selection—describe results of search and selection; ideally present in a flow diagram.	Section 3.1 Search results; Figure 1 (PRISMA flow diagram)	
Results	16b	Cite studies excluded at full-text with reasons.	Appendix C (Figure A1 Full-text articles excluded with primary reason)	
Results	17	Study characteristics—cite characteristics for each included study.	Section 3.3 Description of Included Studies; Appendix F (Table A11)	
Results	18	Risk of bias in studies—present assessments for each included study.	Section 3.2 Study Quality and Risk-of-Bias Assessment; Appendix D (Table A3, Table A4 and Table A5)	
Results	19	Results of individual studies—present results for all outcomes considered for each study.	Results subsections (Pain intensity, Functionality, etc.); Appendix F	
Results	20a–d	Results of syntheses—summarize syntheses; explore heterogeneity; sensitivity analyses; risk of bias on syntheses.	Results (narrative by domain); Discussion (heterogeneity)	Meta-analysis not conducted (not applicable).
Results	21	Reporting biases—present assessments of risk of bias due to missing results.	Appendix E (Publication bias: ‘Unclear’)	Not formally assessed; acknowledged in certainty tables.
Results	22	Certainty of evidence—present assessments of certainty for each outcome.	Section 3.2 (GRADE summary); Appendix E (SoF and Evidence Profiles)	
Discussion	23a	Provide a general interpretation of the results in the context of other evidence.	Section 4. Discussion (opening paragraphs)	
Discussion	23b	Discuss limitations of the evidence included.	Section 4. Discussion (risk of bias/heterogeneity/small samples)	
Discussion	23c	Discuss limitations of the review processes used.	Section 4. Discussion (limitations paragraph: databases only; no gray literature/registries; no automation)	
Discussion	23d	Discuss implications of the results for practice, policy, and future research.	Section 4. Discussion (implications and future directions)	
Other information	24a–c	Registration and protocol—provide registration information; protocol access; amendments.	Section 2.1 Protocol Registration (PROSPERO CRD420251062922); no amendments	
Other information	25	Support—describe sources of financial or non-financial support.	Funding: ‘no external funding’	
Other information	26	Competing interests—declare any competing interests.	Conflicts of Interest: ‘none declared’	
Other information	27	Availability of data, code, and other materials.	Data Availability Statement (‘All data are in the article/Supplementary Information’)	

## Appendix B. Full-Text Articles Excluded with Primary Reason

**Table A2:** Detailed list articles excluded with primary reason (PRISMA 2020, Item 16b).

N	Author	Year	Title	Journal	DOI/PMID	Primary Reason (PRISMA)
1	Caverius U, Åkerblom S, Lexell J, Fischer MR.	2025	Characteristics of Children With Persistent Pain and Their Parents in a Tertiary Interdisciplinary Pain Clinic.	Paediatr Neonatal Pain.	10.1002/pne2.70005	No PNE component
2	Bogard I, Ayre J, Smith J, Pate JW, Sortwell A, Gorringe J, Gordon G, Kamper SJ, Yamato TP.	2024	Exploring Adolescents’ Understanding, Experiences and Beliefs About Pain: A Qualitative Study.	Health Expect	10.1111/hex.70132	No PNE component
3	Geremek A, Ruby L, Lindner C, Niederberger U, Schild U, Jung M, Soyka O, Siniatchkin M.	2023	Child and adolescent psychiatry staff’s knowledge on pain management.	Clin Child Psychol Psychiatry	10.1177/13591045221125334	No PNE component
4	Wakefield EO, Belamkar V, Litt MD, Puhl RM, Zempsky WT.	2022	“There’s Nothing Wrong With You”: Pain-Related Stigma in Adolescents With Chronic Pain.	J Pediatr Psychol.	10.1093/jpepsy/jsab122	No PNE component
5	Foxen-Craft E, Bourchtein E, Kaplan C, Clauw DJ, Scott E.	2023	Pain Widespreadedness, and Not Primary Pain Location, is Associated With Comorbid Symptoms in Children With Chronic Pain.	Clin J Pain.	10.1097/AJP.0000000000001083	No PNE component
6	Bale P, Easton V, Bacon H, Jerman E, Watts L, Barton G, Clark A, Armon K, MacGregor AJ.	2019	The effectiveness of a multidisciplinary intervention strategy for the treatment of symptomatic joint hypermobility in childhood: a randomised, single Centre parallel group trial (The Bendy Study).	Pediatr Rheumatol Online J.	10.1186/s12969-018-0298-x	No PNE component
7	Korterink JJ, Ockeloen LE, Hilbink M, Benninga MA, Deckers-Kocken JM.	2016	Yoga Therapy for Abdominal Pain-Related Functional Gastrointestinal Disorders in Children: A Randomized Controlled Trial.	J Pediatr Gastroenterol Nutr.	10.1097/MPG.0000000000001230	No PNE component
8	Ahlqwist A, Hagman M, Kjellby-Wendt G, Beckung E.	2008	Physical therapy treatment of back complaints on children and adolescents.	Spine (Phila Pa 1976).	10.1097/BRS.0b013e318182c347	No PNE component
9	Sikka I, Chawla C, Seth S, Alghadir AH, Khan M.	2020	Effects of Deep Cervical Flexor Training on Forward Head Posture, Neck Pain, and Functional Status in Adolescents Using Computer Regularly.	Biomed Res Int.	10.1155/2020/8327565	No PNE component
10	Vidal J, Borràs PA, Ponseti FJ, Cantallops J, Ortega FB, Palou P.	2013	Effects of a postural education program on school backpack habits related to low back pain in children.	Eur Spine J.	10.1007/s00586-012-2558-7	No PNE component
11	Vidal J, Borras PA, Ortega FB, Cantallops J, Ponseti X, Palou P.	2011	Effects of postural education on daily habits in children.	Int J Sports Med.	10.1055/s-0030-1270469	No PNE component
12	Dolphens M, Cagnie B, Danneels L, De Clercq D, De Bourdeaudhuij I, Cardon G.	2011	Long-term effectiveness of a back education programme in elementary schoolchildren: an 8-year follow-up study.	Eur Spine J.	10.1007/s00586-011-1856-9	No PNE component
13	Geldhof E, Cardon G, De Bourdeaudhuij I, De Clercq D.	2007	Back posture education in elementary schoolchildren: a 2-year follow-up study.	Eur Spine J.	10.1007/s00586-006-0227-4	No PNE component
14	Pate JW, Harrison LE, Hess CW, Moseley GL, Rush G, Heathcote LC, Simons LE.	2023	Targeting Pain Science Education in Youth With Chronic Pain: What Are the Sticking Points for Youth and Their Parents?	Clin J Pain.	10.1097/AJP.0000000000001088	No PNE component
15	Morris MC, Bruehl S, Stone AL, Garber J, Smith C, Palermo TM, Walker LS.	2021	Does Quantitative Sensory Testing Improve Prediction of Chronic Pain Trajectories? A Longitudinal Study of Youth With Functional Abdominal Pain Participating in a Randomized Controlled Trial of Cognitive Behavioral Treatment.	Clin J Pain.	10.1097/AJP.0000000000000956	No PNE component
16	Hill JJ, Keating JL.	2015	Daily exercises and education for preventing low back pain in children: cluster randomized controlled trial.	Phys Ther.	10.2522/ptj.20140273	No PNE component
17	Andrews NE, Ireland D, Vijayakumar P, Burvill L, Hay E, Westerman D, Rose T, Schlumpf M, Strong J, Claus A.	2023	Acceptability of a Pain History Assessment and Education Chatbot (Dolores) Across Age Groups in Populations With Chronic Pain: Development and Pilot Testing.	JMIR Form Res.	10.2196/47267	No PNE component
18	Kashikar-Zuck S, Ting TV, Arnold LM, Bean J, Powers SW, Graham TB, Passo MH, Schikler KN, Hashkes PJ, Spalding S, Lynch-Jordan AM, Banez G, Richards MM, Lovell DJ.	2012	Cognitive behavioral therapy for the treatment of juvenile fibromyalgia: a multisite, single-blind, randomized, controlled clinical trial.	Arthritis Rheum.	10.1002/art.30644	No PNE component
19	Nilsson S, Wallbing U, Alfvén G, Dalenius K, Fors A, Golsäter M, Rosvall PÅ, Wigert H, Lundberg M.	2019	Development of the Help Overcoming Pain Early (HOPE) Programme Built on a Person-Centred Approach to Support School Nurses in the Care of Adolescents with Chronic Pain-A Feasibility Study.	Children (Basel)	10.3390/children6090095	No PNE component
20	Kashikar-Zuck S, Sil S, Lynch-Jordan AM, Ting TV, Peugh J, Schikler KN, Hashkes PJ, Arnold LM, Passo M, Richards-Mauze MM, Powers SW, Lovell DJ.	2013	Changes in pain coping, catastrophizing, and coping efficacy after cognitive-behavioral therapy in children and adolescents with juvenile fibromyalgia.	J Pain.	10.1016/j.jpain.2012.12.019	No PNE component
21	Mankelow J, Ryan CG, Skidmore N, Potter J, Ravindran D, Chattle R, Browne S, Suri S, Graham A, Pate JW, Newport R, Langford T, Martin D.	2025	An evaluation of a one-day pain science education event in a 16–18 years school setting targeting pain-related beliefs, knowledge, and behavioural intentions: A mixed-methods, non-randomised controlled trial.	Musculoskelet Sci Pract	10.1016/j.msksp.2025.103385.	Healthy participants
22	Mankelow J, Ravindran D, Graham A, Suri S, Pate JW, Ryan CG, Martin D.	2023	An evaluation of a one-day pain science education event in a high school setting targeting pain related beliefs, knowledge, and behavioural intentions.	Musculoskelet Sci Pract.	10.1016/j.msksp.2023.102818	Healthy participants
23	Rheel E, Ickmans K, Wauters A, Van Ryckeghem DML, Barbé K, Malfliet A, Vervoort T.	2022	The Effect of a Pain Educational Video Upon Child Pain-Related Memory and the Moderating Role of Parental Pain- and Non-Pain-Attending Verbalizations: An Experimental Lab-Based Study.	J Pediatr Psychol	10.1093/jpepsy/jsac044	Healthy participants
24	Bacardit Pintó P, Ickmans K, Rheel E, Iwens M, Meeus M, Nijs J, Pas R.	2021	Do Parental Pain Knowledge, Catastrophizing, and Hypervigilance Improve Following Pain Neuroscience Education in Healthy Children?	Children (Basel).	10.3390/children8050420	Healthy participants
25	Martí L, Castarlenas E, Solé E, de la Vega R, Miró J.	2021	Video-based Pain Education in Schools: A Study With Adolescents.	Clin J Pain.	10.1097/AJP.0000000000000906	Healthy participants
26	Louw A, Podalak J, Zimney K, Schmidt S, Puentedura EJ.	2018	Can pain beliefs change in middle school students? A study of the effectiveness of pain neuroscience education.	Physiother Theory Pract	10.1080/09593985.2017.1423142	Healthy participants
27	Cardon GM, de Clercq DL, Geldhof EJ, Verstraete S, de Bourdeaudhuij IM.	2007	Back education in elementary schoolchildren: the effects of adding a physical activity promotion program to a back care program.	Eur Spine J.	10.1007/s00586-006-0095-y	Healthy participants
28	Dullien S, Grifka J, Jansen P.	2018	Cluster-randomized, controlled evaluation of a teacher led multi factorial school based back education program for 10 to 12-year old children.	BMC Pediatr.	10.1186/s12887-018-1280-y	Healthy participants
29	Louw A, Louw C, Podalak J, Zimney K, DeLorenzo J, Maiers N, Puentedura EJ, Mintken P.	2023	Pain Neuroscience Education in Elementary and Middle Schools.	Pediatr Phys Ther.	10.1097/PEP.0000000000001018	Healthy participants
30	Rheel E, Ickmans K, Wauters A, Van Ryckeghem DML, Malfliet A, Vervoort T.	2021	The effect of a pain educational video intervention upon child pain-related outcomes: A randomized controlled study.	Eur J Pain.	10.1002/ejp.1822	Healthy participants
31	Pas R, Meeus M, Malfliet A, Baert I, Oosterwijck SV, Leysen L, Nijs J, Ickmans K.	2018	Development and feasibility testing of a Pain Neuroscience Education program for children with chronic pain: treatment protocol.	Braz J Phys Ther.	10.1016/j.bjpt.2018.02.004	Healthy participants
32	Ciolan F, Bertoni G, Crestani M, Falsiroli Maistrello L, Coppola I, Rossettini G, Battista S.	2025	Perceived factors influencing the success of pain neuroscience education in chronic musculoskeletal pain: a meta-synthesis of qualitative studies.	Disabil Rehabil.	10.1080/09638288.2024.2398141	Ineligible study design
33	Thong HPA, Mardon AK, Evans S.	2025	Pelvic pain education—A short review on pelvic pain and endometriosis educational programs for adolescents.	Aust N Z J Obstet Gynaecol.	10.1111/ajo.13856	Ineligible study design
34	Rezende J, Acalantis L, Nogueira LC, Meziat-Filho N, Ickmans K, Reis FJJ.	2024	Contents and delivery methods of pain neuroscience education in pediatrics: A scoping review.	Musculoskelet Sci Pract.	10.1016/j.msksp.2024.103182	Ineligible study design
35	Kara OK, Gursen C, Ickmans K, Rheel E, Elma O, Cetin SY, Dogan M, Kutluk MG, Kara K.	2024	Enhancing pediatric pain management in Turkey: A modified Delphi study on culturally adapted pain neuroscience education for chronic pain in children.	J Pediatr Nurs.	10.1016/j.pedn.2024.09.001	Ineligible study design
36	Berryman C, Starr T, Ferencz N, Coakley R.	2024	Co-creation in healthcare and research to improve service delivery for young people with chronic pain.	Front Med (Lausanne).	10.3389/fmed.2024.1431155	Ineligible study design
37	Rodriguez-Restrepo A, AuBuchon JD.	2024	Chronic pain in pediatric patients: epidemiology, pathophysiology, and mitigation strategies.	Curr Opin Anaesthesiol.	10.1097/ACO.0000000000001372	Ineligible study design
38	GBD 2021 Diseases and Injuries Collaborators.	2024	Global incidence, prevalence, years lived with disability (YLDs), disability-adjusted life-years (DALYs), and healthy life expectancy (HALE) for 371 diseases and injuries in 204 countries and territories and 811 subnational locations, 1990–2021: a systematic analysis for the Global Burden of Disease Study 2021.	Lancet.	10.1016/S0140-6736(24)00757-8	Ineligible study design
39	GBD 2021 Causes of Death Collaborators.	2024	Global burden of 288 causes of death and life expectancy decomposition in 204 countries and territories and 811 subnational locations, 1990–2021: a systematic analysis for the Global Burden of Disease Study 2021.	Lancet.	10.1016/S0140-6736(24)00367-2	Ineligible study design
40	Schwerdt H, Christe G, Pate JW, Blake C, Smart KM.	2024	The prevalence of chronic pain in adolescents in Central Switzerland: A cross-sectional school-based study protocol.	PLoS One.	10.1371/journal.pone.0297088	Ineligible study design
41	Borucki AN, Benki CM, Peterson EE.	2024	Terminology for discussing chronic pain: Using metaphors to educate families on chronic pediatric pain.	J Pediatr Gastroenterol Nutr.	10.1002/jpn3.12072	Ineligible study design
42	Lin LH, Lin TY, Chang KV, Wu WT, Özçakar L.	2024	Pain neuroscience education for reducing pain and kinesiophobia in patients with chronic neck pain: A systematic review and meta-analysis of randomized controlled trials.	Eur J Pain.	10.1002/ejp.2182	Ineligible study design
43	Allaire C, Yong PJ, Bajzak K, Jarrell J, Lemos N, Miller C, Morin M, Nasr-Esfahani M, Singh SS, Chen I.	2024	Guideline No. 445: Management of Chronic Pelvic Pain	J Obstet Gynaecol Can.	10.1016/j.jogc.2023.102283	Ineligible study design
44	Darnall BD, Edwards KA, Courtney RE, Ziadni MS, Simons LE, Harrison LE.	2023	Innovative treatment formats, technologies, and clinician trainings that improve access to behavioral pain treatment for youth and adults.	Front Pain Res (Lausanne).	10.3389/fpain.2023.1223172	Ineligible study design
45	Shaygan M, Jaberi A, Razavizadegan M, Shayegan Z.	2023	Prevalence of chronic pain and contributing factors: a cross-sectional population-based study among 2379 Iranian adolescents.	Korean J Pain.	10.3344/kjp.22336	Ineligible study design
46	Leake HB, Moseley GL, Murphy LK, Murray CB, Palermo TM, Heathcote LC.	2023	How does pain work? A qualitative analysis of how young adults with chronic pain conceptualize the biology of pain.	Eur J Pain.	10.1002/ejp.2069	Ineligible study design
47	Miró J, Solé E, Castarlenas E, Ingelmo P, Nolla MDC, Escribano J, Reinoso-Barbero F.	2023	The Treatment of Pediatric Pain in Spain: A Survey Study.	Int J Environ Res Public Health.	10.3390/ijerph20032484	Ineligible study design
48	Dale CM, Cioffi I, Novak CB, Gorospe F, Murphy L, Chugh D, Watt-Watson J, Stevens B.	2023	Continuing professional development needs in pain management for Canadian health care professionals: A cross sectional survey.	Can J Pain.	10.1080/24740527.2022.2150156	Ineligible study design
49	Ickmans K, Rheel E, Rezende J, Reis FJJ.	2022	Spreading the word: pediatric pain education from treatment to prevention.	Arch Physiother.	10.1186/s40945-022-00151-4	Ineligible study design
50	Baerg K, Tupper SM, Chu LM, Cooke N, Dick BD, Doré-Bergeron MJ, Findlay S, Ingelmo PM, Lamontagne C, Mesaroli G, Oberlander TF, Poolacherla R, Spencer AO, Stinson J, Finley GA.	2022	Canadian surveillance study of complex regional pain syndrome in children.	Pain.	10.1097/j.pain.0000000000002482	Ineligible study design
51	Hurley-Wallace AL, Nowotny E, Schoth DE, Liossi C.	2021	Online multidisciplinary interventions for paediatric chronic pain: A content analysis.	Eur J Pain.	10.1002/ejp.1827	Ineligible study design
52	Thacker L, Walsh RM, Shinyoung Song G, Khan HA, Parmar P, Vance KT, Grant G, Mesaroli G, Hunter J, Vader K.	2021	Exploring physiotherapy practice within hospital-based interprofessional chronic pain clinics in Ontario.	Can J Pain.	10.1080/24740527.2021.1905508	Ineligible study design
53	Salvat I, Adillón C, Andrés EM, Monterde S, Miró J.	2021	Development of the Conceptualization of Pain Questionnaire: A Measure to Study How Children Conceptualize Pain.	Int J Environ Res Public Health	10.3390/ijerph18073821	Ineligible study design
54	Mueri K, Kennedy M, Pavlova M, Jordan A, Lund T, Neville A, Belton J, Noel M.	2021	The sociocultural context of pediatric pain: an examination of the portrayal of pain in children’s popular media.	Pain	10.1097/j.pain.0000000000002086	Ineligible study design
55	Miró J, Micó JA, Reinoso-Barbero F.	2021	The management of pediatric chronic pain in Spain: a web-based survey study.	Curr Med Res Opin.	10.1080/03007995.2020.1854208	Ineligible study design
56	Emerson ND, Bursch B.	2020	Communicating with Youth about Pain: Developmental Considerations.	Children (Basel).	10.3390/children7100184	Ineligible study design
57	Koechlin H, Locher C, Prchal A.	2020	Talking to Children and Families about Chronic Pain: The Importance of Pain Education-An Introduction for Pediatricians and Other Health Care Providers.	Children (Basel).	10.3390/children7100179	Ineligible study design
58	Pack R MPT, OCS, Gilliland R PhD, Mecham A DPT.	2020	The treatment of central sensitization in an adolescent using pain neuroscience education and graded exposure to activity: A case report.	Physiother Theory Pract.	10.1080/09593985.2018.1551454	Ineligible study design
59	Velazquez Cardona C, Rajah C, Mzoneli YN, Friedrichsdorf SJ, Campbell F, Cairns C, Rodseth RN.	2019	An audit of paediatric pain prevalence, intensity, and treatment at a South African tertiary hospital.	Pain Rep.	10.1097/PR9.0000000000000789	Ineligible study design
60	Leake HB, Heathcote LC, Simons LE, Stinson J, Kamper SJ, Williams CM, Burgoyne LL, Craigie M, Kammers M, Moen D, Pate JW, Szeto K, Moseley GL.	2019	Talking to Teens about Pain: A Modified Delphi Study of Adolescent Pain Science Education.	Can J Pain	10.1080/24740527.2019.1682934	Ineligible study design
61	Kanstrup M, Jordan A, Kemani MK.	2019	Adolescent and Parent Experiences of Acceptance and Commitment Therapy for Pediatric Chronic Pain: An Interpretative Phenomenological Analysis.	Children (Basel).	10.3390/children6090101	Ineligible study design
62	Grasaas E, Fegran L, Helseth S, Stinson J, Martinez S, Lalloo C, Haraldstad K.	2019	iCanCope With Pain: Cultural Adaptation and Usability Testing of a Self-Management App for Adolescents With Persistent Pain in Norway.	JMIR Res Protoc.	10.2196/12940	Ineligible study design
63	Robins H, Perron V, Heathcote LC, Simons LE.	2016	Pain Neuroscience Education: State of the Art and Application in Pediatrics.	Children (Basel).	10.3390/children3040043	Ineligible study design
64	Manworren RC, Stinson J.	2016	Pediatric Pain Measurement, Assessment, and Evaluation.	Semin Pediatr Neurol.	10.1016/j.spen.2016.10.001	Ineligible study design
65	Louw A, Zimney K, Puentedura EJ, Diener I.	2016	The efficacy of pain neuroscience education on musculoskeletal pain: A systematic review of the literature.	Physiother Theory Pract.	10.1080/09593985.2016.1194646	Ineligible study design
66	Carr EC, Briggs EV, Briggs M, Allcock N, Black P, Jones D.	2016	Understanding factors that facilitate the inclusion of pain education in undergraduate curricula: Perspectives from a UK survey.	Br J Pain.	10.1177/2049463716634377	Ineligible study design
67	Maciel SC, Jennings F, Jones A, Natour J.	2009	The development and validation of a Low Back Pain Knowledge Questionnaire—LKQ.	Clinics (Sao Paulo).	10.1590/S1807-59322009001200006	Ineligible study design
68	Zhang Y, Yang C.	2024	Influence of pain neuroscience education and exercises for the management of neck pain: A meta-analysis of randomized controlled trials.	Medicine (Baltimore).	10.1097/MD.0000000000040760	Ineligible study design
69	Harrison LE, Pate JW, Richardson PA, Ickmans K, Wicksell RK, Simons LE.	2019	Best-Evidence for the Rehabilitation of Chronic Pain Part 1: Pediatric Pain.	J Clin Med.	10.3390/jcm8091267	Ineligible study design
70	Clinch J, Eccleston C.	2009	Chronic musculoskeletal pain in children: assessment and management.	Rheumatology (Oxford).	10.1093/rheumatology/kep001	Ineligible study design
71	O’Sullivan K, O’Keeffe M, Forster BB, Qamar SR, van der Westhuizen A, O’Sullivan PB.	2019	Managing low back pain in active adolescents.	Best Pract Res Clin Rheumatol.	10.1016/j.berh.2019.02.005	Ineligible study design
72	Fechner R, Verhagen A, Alcock M, Norton J, Stubbs PW, Harrison LE, Pate JW.	2024	The Effectiveness of Pain Science Education on Caregiver and Children’s Knowledge, Beliefs, Attitudes, and Behaviors-A Systematic Review and Meta-Analysis.	J Pain.	10.1016/j.jpain.2024.104578	Ineligible study design
73	Barrett MJ, Barnett PL.	2016	Complex Regional Pain Type 1.	Pediatr Emerg Care.	10.1097/PEC.0000000000000731	Ineligible study design
74	Lalloo C, Jibb LA, Rivera J, Agarwal A, Stinson JN.	2015	There’s a Pain App for That: Review of Patient-targeted Smartphone Applications for Pain Management.	Clin J Pain.	10.1097/AJP.0000000000000171	Ineligible study design
75	de la Vega R, Miró J.	2024	mHealth: a strategic field without a solid scientific soul. a systematic review of pain-related apps.	PLoS One.	10.1371/journal.pone.0101312	Ineligible study design
76	Anyachukwu CC, Amarah CC, Atueyi BC, Anthony I, Nweke M, Abaraogu U.	2024	Effectiveness of Back care education Programme among school children: a systematic review of randomized controlled trials.	BMC Pediatr.	10.1186/s12887-024-04563-y	Ineligible study design
77	Yu H, Southerst D, Wong JJ, Verville L, Connell G, Ead L, Mior S, Hestbaek L, Swain M, Brunton G, Shearer HM, Papaconstantinou E, To D, Germann D, Pohlman K, Cedraschi C, Cancelliere C.	2024	Rehabilitation of back pain in the pediatric population: a mixed studies systematic review.	Chiropr Man Therap.	10.1186/s12998-024-00538-z	Ineligible study design
78	Klausen SH, Rønde G, Tornøe B, Bjerregaard L.	2019	Nonpharmacological Interventions Addressing Pain, Sleep, and Quality of Life in Children and Adolescents with Primary Headache: A Systematic Review.	J Pain Res.	10.2147/JPR.S216807	Ineligible study design
79	van der Velde G, Yu H, Paulden M, Côté P, Varatharajan S, Shearer HM, Wong JJ, Randhawa K, Southerst D, Mior S, Sutton D, Jacobs C, Taylor-Vaisey A.	2016	Which interventions are cost-effective for the management of whiplash-associated and neck pain-associated disorders? A systematic review of the health economic literature by the Ontario Protocol for Traffic Injury Management (OPTIMa) Collaboration.	Spine J.	10.1016/j.spinee.2015.08.025	Ineligible study design
80	Liegl G, Boeckle M, Leitner A, Pieh C.	2016	A meta-analytic review of brief guided self-help education for chronic pain.	Eur J Pain.	10.1002/ejp.881	Ineligible study design
81	Michaleff ZA, Kamper SJ, Maher CG, Evans R, Broderick C, Henschke N.	2014	Low back pain in children and adolescents: a systematic review and meta-analysis evaluating the effectiveness of conservative interventions.	Eur Spine J.	10.1007/s00586-014-3461-1	Ineligible study design
82	García-Moreno JM, Calvo-Muñoz I, Gómez-Conesa A, López-López JA.	2025	Therapeutic Exercise is Effective in Reducing the Intensity of Nonspecific Low Back Pain in Children and Adolescents: A Systematic Review and Network Meta-analysis.	Arch Phys Med Rehabil.	10.1016/j.apmr.2024.11.002.	Ineligible study design
83	Dobe, M; Zernikow, B	2019	Inpatient Pain Treatment: Module 4 (Integrating the Family System)	springer nature	10.1007/978-3-030-19201-3_11	Ineligible study design
84	Serena Maria Dib, Gaelle Rached, Dimitri Fiani, Souraya Torbey,	2021	The Role of Pain Education in the Treatment of Pediatric Chronic Pain	J. Am. Acad. Child Adolesc. Psychiatry	10.1016/j.jaac.2021.09.395	Ineligible study design
85	Shankey-Smith, W	2025	Paediatric chronic pain	Anaesth. Intensive Care Med.	10.1016/j.mpaic.2025.04.013	Ineligible study design
86	Palahí-Calsina, I., Jubany, J., Sordo, L., Lorente, S., Espelt, A. y Borao, O.	2024	Effectiveness of pain neuroscience education among adults with chronic neck pain. Systematic review	Eur. J. Physiother.	10.1080/21679169.2024.2365694	Ineligible study design
87	Stinson JN, Lalloo C, Harris L, Isaac L, Campbell F, Brown S, Ruskin D, Gordon A, Galonski M, Pink LR, Buckley N, Henry JL, White M, Karim A.	2014	iCanCope with Pain™: User-centred design of a web- and mobile-based self-management program for youth with chronic pain based on identified health care needs.	Pain Res Manag.	10.1155/2014/935278	Ineligible study design
88	Sil S, Arnold LM, Lynch-Jordan A, Ting TV, Peugh J, Cunningham N, Powers SW, Lovell DJ, Hashkes PJ, Passo M, Schikler KN, Kashikar-Zuck S.	2014	Identifying treatment responders and predictors of improvement after cognitive-behavioral therapy for juvenile fibromyalgia.	Pain.	10.1016/j.pain.2014.03.005	Ineligible study design
89	Murray CB, de la Vega R, Loren DM, Palermo TM.	2020	Moderators of Internet-Delivered Cognitive-Behavioral Therapy for Adolescents With Chronic Pain: Who Benefits From Treatment at Long-Term Follow-Up?	J Pain.	10.1016/j.jpain.2019.10.001	Ineligible study design

## Appendix C. Full Electronic Search Strategies

Registration: CRD420251062922

Date last searched: 21 July 2025.

## Appendix D. Results of Risk of Bias

**Table A3:** Risk of bias. RoB-2.

Study	Variable	D1a	D1b	D2	D3	D4	D5	Overall
		Randomization	Cluster Trial Part b	Deviations	Missing Data	Measurement	Reporting	
Palermo [39]	Child—NRS-11 (pain intensity)	Low risk	Not applicable	Low risk	Low risk	Low risk	Low risk	Low risk
	Child—BAPQ (anxiety)	Low risk	Not applicable	Low risk	Low risk	Low risk	Low risk	Low risk
	Child—CALI (disability)	Low risk	Not applicable	Low risk	Low risk	Low risk	Low risk	Low risk
	Child—ASWS (sleep)	Low risk	Not applicable	Low risk	Low risk	Low risk	Low risk	Low risk
	Child—HHI-Pain (helplessness)	Low risk	Not applicable	Low risk	Low risk	Low risk	Low risk	Low risk
	Child—TEI-SF (satisfaction)	Low risk	Not applicable	Low risk	Low risk	Low risk	Low risk	Low risk
	Parent—BAPQ-PIQ (anxiety)	Low risk	Not applicable	Low risk	Low risk	Low risk	Low risk	Low risk
	Parent—ARCS (protective behavior)	Low risk	Not applicable	Low risk	Low risk	Low risk	Low risk	Low risk
	Parent—HHI-Pain (helplessness)	Low risk	Not applicable	Low risk	Low risk	Low risk	Low risk	Low risk
	Parent—TEI-SF (satisfaction)	Low risk	Not applicable	Low risk	Low risk	Low risk	Low risk	Low risk
Andías [40]	EVA (pain intensity)	Some concerns	Not applicable	Some concerns	Low risk	High risk	Low risk	Some concerns
	Muscle Assesment	Some concerns	Not applicable	Low risk	Low risk	High risk	Low risk	Some concerns
	STAIC (anxiety)	Some concerns	Not applicable	Low risk	Low risk	High risk	Low risk	Some concerns
	PCS (catastrophizing)	Some concerns	Not applicable	Low risk	Low risk	High risk	Low risk	Some concerns
	NPQ (knowledge)	Some concerns	Not applicable	Low risk	Low risk	High risk	Low risk	Some concerns
	PGIC (satisfaction)	Some concerns	Not applicable	Low risk	Low risk	Some concerns	Low risk	Some concerns
Pas [41]	Child—FPS-R (pain intensity)	Low risk	Not applicable	High risk	Low risk	Low risk	Low risk	Some concerns
	Parents—PCS-P (catastrophizing)	Low risk	Not applicable	High risk	Low risk	Low risk	Low risk	Some concerns
	Parents—FDI-P (function)	Low risk	Not applicable	High risk	Low risk	Low risk	Low risk	Some concerns
	Parents—FOPQ-P (fear of pain)	Low risk	Not applicable	High risk	Low risk	Low risk	Low risk	Some concerns
Louw [42]	rNPQ (knowledge)	Low risk	Some concerns	Low risk	Some concerns	High risk	Some concerns	High risk
	FABQ-PA (Kinesiophobia)	Low risk	Some concerns	Low risk	Some concerns	High risk	Some concerns	High risk
	Behavior change	Low risk	Some concerns	Low risk	Some concerns	High risk	Some concerns	High risk
	Medication use	Low risk	Some concerns	Low risk	Some concerns	High risk	Some concerns	High risk
Kisling [43]	NRS (pain intensity)	Some concerns	Low risk	Low risk	Low risk	High risk	Low risk	Some concerns
	PKQ-CH (knowledge)	Some concerns	Low risk	Low risk	Some concerns	High risk	Some concerns	High risk
	PPCI-r (passive coping)	Some concerns	Low risk	Low risk	Some concerns	Low risk	Some concerns	Some concerns
	PPDI (disability)	Some concerns	Low risk	Low risk	Some concerns	Low risk	Some concerns	Some concerns
Walker [44]	CCSSI-24 and API (pain intensity)	Low risk	Not applicable	Low risk	Low risk	Low risk	Low risk	Low risk
	TEI-SF (satisfaction)	Low risk	Not applicable	Low risk	Low risk	Low risk	Low risk	Low risk
Andías [45]	NPQ (knowledge)	Low risk	Not applicable	Low risk	Low risk	High risk	Some concerns	High risk
	PCS (catastrophizing)	Low risk	Not applicable	Low risk	Low risk	Low risk	Some concerns	Some concerns
	TSK (kinesiophobia)	Low risk	Not applicable	Low risk	Low risk	Low risk	Some concerns	Some concerns
	FDI (function)	Low risk	Not applicable	Low risk	Low risk	Low risk	Some concerns	Some concerns
	CSES (self-efficacy)	Low risk	Not applicable	Low risk	Low risk	Low risk	Some concerns	Some concerns
	BaSIQS (insomnia)	Low risk	Not applicable	Low risk	Low risk	Low risk	Some concerns	Some concerns
	CSI (Central Sensitizacion)	Low risk	Not applicable	Low risk	Low risk	Low risk	Low risk	Low risk
	PGIC (Patient Global Impression of Change)	Low risk	Not applicable	Low risk	Low risk	Low risk	Low risk	Low risk
Beach [46]	COPI (knowledge)	High risk	Not applicable	Low risk	Some concerns	Low risk	Low risk	High risk
Menes [47]	COPAQ (knowledge)	Some concerns	Not applicable	Some concerns	Some concerns	Low risk	Low risk	Some concerns

**Table A4:** Risk of bias. CAS.

CASP Criteria	Neto [48]	
	Variable	
	Relevance of Knowledge	Intervention Adequacy
1. Clear Study Aims	✅ Fully addressed	✅ Fully addressed
2. Appropriate Methodology	✅ Thematic analysis	✅ Thematic analysis
3. Research Design	⚠️ Single-center	⚠️ Single-center
4. Recruitment Strategy	⚠️ Homogeneous sample	⚠️ Homogeneous sample
5. Data Collection	✅ Rigorous (recordings)	✅ Rigorous (recordings)
6. Researcher-Participant Relationship	⚠️ Potential bias (therapists as researchers)	⚠️ Potential bias (therapists as researchers)
7. Ethical Considerations	✅ Approved + consent	✅ Approved + consent
8. Data Analysis	✅ Triangulation	✅ Triangulation
9. Findings	✅ Rich quotes + quant data	✅ Rich quotes + quant data
10. Research Value	✅ Novel insights	✅ Novel insights
11. Overall Rating	Excellent	Excellent

**Table A5:** Risk of Bias. NIH Quality Assessment Tool.

Wager [49]		
Item	NIH Tool Question	Assessment
1	Was the research question or objective clearly stated?	Yes
2	Were eligibility/selection criteria for the study population prespecified and applied uniformly?	Partially
3	Was the study population representative of the intended population?	Unclear
4	Were all participants clearly described at each stage of the study?	Yes
5	Was the intervention clearly described and delivered consistently to all participants?	Yes
6	Were outcome measures assessed more than once before the intervention?	No
7	Were outcome measures assessed more than once after the intervention?	No
8	Were outcome measures clearly defined, valid, and reliable?	Partially
9	Were the outcome assessors blinded to the intervention status of participants?	No
10	Was loss to follow-up after baseline 20% or less?	Yes
11	Were all participants who were enrolled included in the analysis?	Yes
12	Were statistical methods appropriate for the study design?	Yes
13	Were potential confounding variables measured and adjusted for?	Partially
14	Are the results believable given the study design and limitations?	Yes, with caution
Overall Quality: Yes: 7; Partially: 3; No count: 3		Fair

## Appendix E. GRADE Summary of Findings and Evidence Profiles (By Informant)

**Table A6:** Summary of Findings (SoF)-Children/adolescents (self-report).

Outcome	Importance	No. of Studies	Direction of Effect	Certainty (GRADE)
Pain knowledge	Critical	7	Consistent and sustained improvement	Moderate
Disability/Function	Critical	4	Modest improvements; heterogeneous	Low
Pain intensity	Critical	6	Mixed results; 2/6 with sustained reduction	Low
Anxiety	Important	2	Minor effects/not sustained	Very low
Catastrophizing	Important	2	Decreases (significant in 1)	Low
Kinesiophobia	Important	2	Improvements in both groups; significant in 1	Low
Sleep	Important	2	Slight improvement; in one RCT, favors CBT	Low
Self-efficacy	Important	1	Improvement in both groups	Very low
Medication use	Important	1	Reduction with PNE + reinforcements	Very low
Patient Global Impression of Change	Critical	1	Perceived improvement post-treatment	Low ^q^
Satisfaction with treatment/care	Important	3	High/moderate; variability across studies	Low ^p r^
Pain coping	Important	1	Small improvements/inconclusive	Very low
Central sensitization	Important	1	Reduction/inconclusive depending on instrument	Very low
Depression	Important	1	Small/not sustained effects	Very low
Miscarried helping	Important	1	Small/moderate reduction (if reported)	Very low
Adverse events	Critical	0	Not reported	— ^u^

Notes. Superscript letters refer to Table A10 (explanations and rules). An asterisk (*) next to a certainty rating indicates downgrading for INDIRECTNESS due to parent proxy measurement (when applicable). Certainty (GRADE): High, Moderate, Low, Very low. Abbreviations: RCT: Randomized Controlled Trial; CBT: Cognitive Behavioral Therapy; PNE: Pain Neuroscience Education.

**Table A7:** Summary of Findings (SoF)-Parents/caregivers (self-report).

Outcome	Importance	No. of Studies	Direction of Effect	Certainty (GRADE)
Parental catastrophizing	Important	1	Small to moderate decreases	Low
Caregiver anxiety/stress	Important	1	Small/unsustained effects	Very low
Parental behaviors/family accommodation	Important	2	Small to moderate reductions; slight heterogeneity	Low
Disability/Function (proxy)	Critical	2	Mixed/inconclusive results	Low * ^ℓ^
Pain intensity (proxy)	Critical	2	Mixed/inconclusive results	Low * ^ℓ^
Satisfaction with treatment/care	Important	2	High/moderate; variability between studies	Very low ^p r^

Notes. ‘(proxy)’ indicates parent-reported outcomes for the child; proxy outcomes were downgraded for INDIRECTNESS. Superscript letters refer to Table A10 (explanations and rules). An asterisk (*) next to a certainty rating indicates downgrading for INDIRECTNESS due to parent proxy measurement (when applicable). Certainty (GRADE): High, Moderate, Low, Very low. ℓ: When a child’s outcome is measured by parent proxy, consider downgrading for INDIRECTNESS.

**Table A8:** Evidence profile—Children/adolescents.

Outcome	Risk of Bias	Inconsistency	Indirectness	Imprecision	Publication Bias	Other Considerations	Certainty
Pain knowledge	Some concerns	Not serious	Not serious	Serious	Unclear	-	Moderate
Disability/Function	Some concerns	Serious	Not serious	Serious	Unclear	-	Low
Pain intensity	Some concerns	Serious	Not serious	Serious	Unclear	-	Low
Anxiety	Low	Serious	Possible (comparators)	Very serious	Unclear	-	Very low
Catastrophizing	Some concerns	Not serious	Not serious	Serious	Unclear	-	Low
Patient Global Impression of Change	Some concerns	Not assessable	Not serious	Serious	Unclear	-	Low
Satisfaction (child/adolescent)	Some concerns (unvalidated measurement)	Serious	Not serious	Serious	Unclear	-	Low
Miscarried helping	Some concerns	Not assessable	Not serious	Very serious	Unclear	-	Very low
Pain coping	Some concerns	Not assessable	Not serious	Very serious	Unclear	-	Very low
Central sensitization	Some concerns	Not assessable	Not serious	Very serious	Unclear	-	Very low
Depression	Low	Not assessable	Not serious	Very serious	Unclear	-	Very low

**Table A9:** Evidence profile—Parents/caregivers.

Outcome	Risk of Bias	Inconsistency	Indirectness	Imprecision	Publication Bias	Other Considerations	Certainty
Parental catastrophizing	Some concerns	Not assessable	Not serious	Serious	Unclear	-	Low
Caregiver anxiety/stress	Low	Serious	Not serious	Very serious	Unclear	-	Very low
Parental behaviors/family accommodation	Some concerns	Unclear	Not serious	Serious	Unclear	Heterogeneous instruments	Low
Satisfaction (parent/caregiver)	Some concerns (measurement unvalidated)	Serious	Not serious	Very serious	Unclear	-	Very low
Disability/Function (proxy)	Some concerns	Serious	Serious (proxy measurement)	Serious	Unclear	-	Low *
Pain intensity (proxy)	Some concerns	Serious	Serious (proxy measurement)	Serious	Unclear	-	Low *

An asterisk (*) next to a certainty rating indicates downgrading for INDIRECTNESS due to parent proxy measurement.

**Table A10:** GRADE Explanations and Notes (downgrading criteria, proxy rules, and definitions).

Code	Description
m	If the program includes a parent-directed component, parent outcomes are direct (do not downgrade for indirectness merely for being parental).
n	Prioritize child self-report for subjective outcomes; use proxy only if self-report is unavailable.
p	Satisfaction measures are heterogeneous and often unvalidated in pediatrics.
q	PGIC is susceptible to expectation/non-blinding; few studies.
r	Distinguish satisfaction with the program/intervention vs. with services/care.
s	Proxy rows are shown only if ≥1 study contributes data; otherwise omitted from SoF and mentioned in text.
t	In the evidence profile, proxy outcomes are downgraded for INDIRECTNESS (≥1 level).
u	Adverse events: critical pre-specified outcome; not reported by included studies (kept as a row in SoF and noted in text).
ℓ	When a child’s outcome is measured by parent proxy, consider downgrading for INDIRECTNESS.

Editorial note: Only the following superscripts are used in the SoF tables: ℓ, p, q, r, u; other codes (e.g., m, n, s, t) are general rules and do not appear as superscripts.

## Appendix F. Characteristics of the Included Studies

**Table A11:** Characteristics of the included studies.

Author	Sample	Intervention	Assessment	Results (GE Versus GC)
Palermo et al. [39]	*n* = 273 11–17 years - GE: 138 - GC: 135 - H: 70/M: 205 Multiple CP Parents included	Randomized controlled trial. Setting: Home. Duration: 4 weeks. Follow-up: 6 months. EG: Internet-delivered CBT. CG: Internet-delivered PNE.	- Intensity: NRS-11 - Emotional function (depression and anxiety subscales): BAPQ - Disability: CALI - Sleep: ASWS - Failed help: HHI-Pain - Satisfaction: TEI-SF - Parents: Emotional function: BAPQ-PIQ Protective behavior: ARCS Failed help: HHI-Pain Satisfaction: TEI-SF	- NRS-11: no significant differences after intervention (*p* = 0.24) or at follow-up (*p* = 0.07). - BAPQ depression subscale: EG showed a significant reduction (p = 0.04). BAPQ anxiety subscale: EG reduction (p = 0.04), but neither effect was maintained at follow-up. - CALI: non-significant reduction in EG at 6 months (p = 0.03). No difference after intervention (p = 0.39). - ASWS: non-significant reduction at 6 months (p = 0.04). No difference after intervention (p = 0.07). - HHI-Pain: significant improvement at 6 months (p = 0.002). - TEI-SF: significantly higher in EG (p < 0.001). - Parents BAPQ-PIQ: small to moderate effects on anxiety (p = 0.02), depression (p = 0.002), maladaptive behaviors (p = 0.001), and self-blame (p = 0.003). - Parents HHI-Pain: no significant differences (p = 0.08). - Parents ARCS: significant reduction in CG through follow-up (p = 0.001). - Parents TEI-SF: significantly higher in EG (p < 0.001).
Andías et al. [40]	*n* = 43 16–18 years - GE: 21 - GC: 22 - H:6(GC) + 9 (GE) = 15/ M:16(GC) + 12(GE) = 28 Cervical CP	Pilot randomized controlled study. Setting: School. Duration: 1 session/week for 4 weeks. Follow-up: Not performed. EG: PNE and exercises. CG: No intervention.	- Intensity: EVA - Muscle assessment: Deep neck flexors Scapular extensors Scapular stabilizers - Anxiety: STAIC - Catastrophizing: PCS - Knowledge: NPQ - Satisfaction: PGIC	- VAS: non-significant decrease in EG (*p* = 0.05). - Muscle assessment: significant increase in scapular stabilizer endurance (*p* = 0.004) in EG. - STAIC: non-significant decrease in EG (*p* = 0.77). - PCS: non-significant decrease in EG (*p* = 0.25). - NPQ: significant improvement in EG (*p* = 0.42). - PGIC: 85.7% of the EG reported a slight change and 47.6% reported a significant improvement, versus 1.8% in the CG.
Neto et al. [48] This trial was conducted after the Andías 2018 study.	*n* = 43 16–18 years - GE: 21 - GC: 22 - H: 9/M: 12 Cervical CP	Qualitative randomized controlled trial. Setting: School. Duration: 1 session/week for 4 consecutive weeks. Follow-up: Not performed. EG: Exercises + PNE. CG: No intervention.	Relevance of the knowledge acquired and perception of the intervention: through 4 focus group interviews analyzed using the COREQ criteria (items 1–4, 9–21, 24–26, and 29–32).	- Perceived relevance of the knowledge acquired: facilitator of changes in feelings, attitudes, and behaviors toward pain, with reduced anxiety, fear, and catastrophizing, and an increased sense of self-efficacy to manage it. - Perception of the appropriateness of the intervention: well received by all participants, useful, and with appropriate materials and strategies.
Wager et al. [49]	*n* = 33 10–15 years - GE = GC - H: 16/M: 16 Multiple CP	Feasibility study. Setting: School. Duration: 45–50 min. Follow-up: Not performed. EG = CG = PNE (10-min educational film on CP management).	- Knowledge: PKQ-CH	- PKQ-CH: significant increase after intervention (*p* < 0.05).
Pas et al. [41]	*n* = 28 6 y 12 years - GE:14 - GC:14 - H: 10/M: 18 FAP Parents included	Comparative pilot randomized study. Setting: University Hospital. Duration: Once per week for 3 weeks. Follow-up: At 3 weeks. EG: 1 session of hypnotherapy + PNE (PNE4Kids). CG: 2 sessions of hypnotherapy.	- Intensity: FPS-R - Parents: Catastrophizing: PCS-P Function: FDI-P Fear of pain: FOPQ-P	- FPS-R: no significant effect in either group (*p* = 0.045). - Parents PCS-P: significant improvements after intervention and at follow-up (*p* < 0.01). - Parents FDI-P: significant improvements after intervention and at follow-up (*p* = 0.014). - Parents FOPQ-P: significant improvements after intervention and at follow-up (*p* = 0.002).
Louw et al. [42]	*n* = 96 11–13 years - G1: 38 - G2:16 - G3:42 - H: -/M: - Multiple CP	Quasi-experimental clinical trial. Setting: School. Duration: 30-min lecture. Follow-up: 6 months. G1: Lecture on PNE. G2: Usual care (UC). G3: PNE + 2 booster sessions (PNEBoost).	Knowledge: rNPQ - Kinesiophobia: FABQ-PA - Behavior change: self-designed survey - Medication use	- rNPQ: significantly higher at 6 months (*p* = 0.017) in G3. - FABQ-PA: no significant differences (*p* = 0.871). - G1 missed fewer school days than G2 (*p* = 0.037). - No differences between groups in school attendance, physical education, recess, sports participation, doctor visits, or rehabilitation visits. - G3 used significantly fewer pain medications in the following school year (*p* = 0.024).
Kisling et al. [43]	*n* = 108 9–14 years - GE: 49 - GC: 59 - H: -/M: - Multiple CP	Randomized controlled trial. Setting: School. Duration: 4–5 weeks. Follow-up: At 4–5 weeks. EG = CG = PNE (10-min educational film on CP management).	- Intensity: NRS - Knowledge: PKQ-CH - Pain Coping: PPCI r - Disability: PPDI	- NRS: main group effect, with change not attributable to the intervention (*p* > 0.05). - PKQ-CH: significant increase in EG (*p* < 0.001). - PPCI-r: no significant change (*p* > 0.05). - PPDI: no significant change (*p* > 0.05).
Walker et al. [44]	*n* = 278 11–17 years - GE: 142 - GC: 136 - H: 94/M: 184 FAP and GI symptoms Parents included	Randomized controlled trial. Setting: Home. Duration: 8 weeks. Follow-up: At 6 and 12 months. EG: Internet-delivered CBT. CG: Internet-delivered PNE.	- Intensity: CSSI-24 y API. - Function: PROMIS PPI vs8 - Satisfaction: TEI-SF	- CSSI-24: significant improvement in both CG and EG, with no differences between groups (*p* > 0.76) when participants were not distinguished by subgroup. - PROMIS: significant improvement in both CG and EG, but no differences between groups (*p* > 0.76) when participants were not distinguished by subgroup. - Greater significant improvement in GI symptoms was observed in patients assigned to the CG–HPD subgroup (high pain, maladaptive profile, poor prognosis) after treatment (*p* = 0.01), maintained at 6 and 12 months (*p* = 0.03). - Satisfaction: significantly higher in EG among both parents and adolescents (*p* < 0.001).
Andías et al. [45]	*n =* 127 15–18 years - GE:68 - GC: 59 - H: -/M: - Cervical CP	Randomized controlled trial. Setting: School. Duration: 8 weeks. Follow-up: At 6 months. EG: Exercise + PNE. CG: Exercise.	- Intensity: NPRS y PPT - Muscle assessment: Deep neck flexors Scapular extensors Scapular stabilizers. - Catastrophizing: PCS - Kinesiophobia: TSK - Function: FDI - Self-efficacy: CSES - Sleep: BaSIQS - Central Sensitization: CSI - Knowledge: NPQ - Patient’s Global Impression of Change: PGIC	- NPRS: significant decrease in both groups at follow-up (*p* < 0.001), with a significant difference in EG (*p* < 0.05). - PPT: significant increase in both groups at follow-up (*p* < 0.001), with a significant difference in EG (*p* < 0.05). - Muscle assessment: significant increase in both groups (*p* < 0.001). - PCS: significant decrease in both groups at follow-up (*p* < 0.001). - TSK: significant decrease in both groups at follow-up (*p* < 0.001). - FDI: significant decrease in disability in both groups at follow-up (*p* < 0.001). - CSES: significant improvement in both groups at follow-up (*p* > 0.05). - BaSIQS: significant improvement in both groups at follow-up (*p* > 0.05). - CSI: significant decrease in CS symptoms in both groups at follow-up (*p* < 0.001). - NPQ: increase in EG (*p* < 0.001). - PGIC: significant improvement in EG (*p* < 0.05).
Beach et al. [46]	*n =* 17 12–18 years - GE = GC - H: 13/M: 4 Headaches Parents included	Pilot randomized controlled study Setting: University Pediatric Neurology Clinic. Duration: 10 min. Follow-up: At 6 months. EG = CG: PNE with a 3D brain model and a 10-min discussion.	- Knowledge: COPI	- COPI: significant increase after the intervention (*p* = 0.002).
Menés et al. [47]	*n* = 73 11–13 years - GE:41 - GC: 32 - H: -/M: - Multiple CD	Randomized controlled trial. Setting: School. Duration: Two sessions with a 4-week interval between them. Follow-up: At 7 and 13 months. EG: PNE. CG: Standard teaching program.	- Knowledge: COPAQ	- COPAQ: significant improvement at follow-up (*p* < 0.001).

ARCS: Protect subscale from the Adult Responses to Children’s Symptoms; ASWS: Adolescent Sleep–Wake Scale; BAPPIQ: Bath Adolescent Pain—Parental Impact Questionnaire; BAPQ: Bath Adolescent Pain Questionnaire; BaSIQS: Basic Scale of Insomnia Complaints and Sleep Quality; CALI: Child Activity Limitations; CDI: Children’s Depression Inventory; COPI: Concept Of Pain Inventory; COPAQ: Pain Conceptualization Questionnaire; CSES: Child Self-Efficacy Scale; CSI: Central Sensitization Inventory; CSSI-24: Children’s Somatic Symptoms Inventory—24; DC: Chronic Pain; EVA: Visual Analog Scale; FABQ-PA: Fear Avoidance Beliefs Questionnaire Physical Activity; FAP: Functional abdominal pain; FDI: Functional Disability Inventory; FDI-P: Functional Disability Inventory-parent proxy; FOPQ-P Fear of Pain Questionnaire-parent report; FPS-R: The Faces Pain Scale-Revised; GE: Experimental Group; GC: Control Group; HHI-Pain: Helping for Health Inventory adapted for chronic pain; IED-P: Functional disability; NPQ: Neurophysiology of Pain Questionnaire; NPRS: Numeric Pain Rating Scale; NRS: Numeric Rating Scale; PBQ-SF: Pain Beliefs Questionnaire-Short Form.; PCS Pain Catastrophizing Scale; PCS-P: Pain Catastrophizing Scale for parents; PGIC: Patient’s Global Impression of Change; PKQ-CH: Pain Knowledge Questionnaire for Children; PNE: Pain Neuroscience Education; PPCI r: Pediatric Pain Coping Inventory—revised; PPDI: Pediatric Pain Disability Index; PPT: Pressure Pain Threshold; PRI: Catastrophizing subscale of the Pain Response Inventory; PROMIS: PROMIS Pediatric Pain Interference-Short Form; SC: Central Sensitization; STAIC: State–Trait Anxiety Inventory for Children; TEI-SF: Treatment Evaluation Inventory, short form; TSK: Tampa Scale for Kinesiophobia.
