# Supplementary material for: Pain Neuroscience Education in Children and Adolescents with Chronic Pain: A Systematic Review
Source: Children (Basel). 2025 Oct 1;12(10):1317. doi: 10.3390/children12101317 (PMC12564171; doi:10.3390/children12101317)
Supplement: Supplementary file 1 [file children-12-01317-s001.zip › children-3853717-supplementary.pdf]

Table S1. Sources of PNE used in the studies

	Source of the PNE			
**Study**	***Explain pain*** [50]	***Therapeutic neuroscience education: teaching patients about pain.*** [51]	***Why Do I Hurt?*** [53]	***Cuentos Analgésicos*** [54]
Palermo et al. [39]	x			
Andias et al. [40]	x	x		
Neto et al. [48]	x	x		
Wager et al. [49]		x		
Pas et al. [41]	x			
Louw et al. [42]	x	x	x	
Kisling et al. [43]		x		
Walker et al. [44]	x			
Andias et al. [45]	x	x		
Beach et al. [46]	x			
Menés [47]	x		x	x
